# Regeneration Enhances Metastasis: A Novel Role for Neurovascular Signaling in Promoting Melanoma Brain Metastasis

**DOI:** 10.3389/fnins.2019.00297

**Published:** 2019-04-09

**Authors:** Roshini Prakash, Sivan Izraely, Nikita S. Thareja, Rex H. Lee, Maya Rappaport, Riki Kawaguchi, Orit Sagi-Assif, Shlomit Ben-Menachem, Tsipi Meshel, Michal Machnicki, Shuichi Ohe, Dave S. Hoon, Giovanni Coppola, Isaac P. Witz, S. Thomas Carmichael

**Affiliations:** ^1^Department of Neurology, David Geffen School of Medicine, University of California, Los Angeles, Los Angeles, CA, United States; ^2^Department of Cell Research and Immunology, School of Molecular Cell Biology and Biotechnology, The George S. Wise Faculty of Life Sciences, Tel Aviv University, Tel Aviv, Israel; ^3^Department of Psychiatry and Biobehavioral Sciences, Semel Institute for Neuroscience and Human Behavior, University of California, Los Angeles, Los Angeles, CA, United States; ^4^Department of Translational Molecular Medicine, John Wayne Cancer Institute at Providence Saint John’s Health Center, Santa Monica, CA, United States

**Keywords:** angiogenesis, stroke, neuroblast, astrocytosis, gliosis

## Abstract

Neural repair after stroke involves initiation of a cellular proliferative program in the form of angiogenesis, neurogenesis, and molecular growth signals in the surrounding tissue elements. This cellular environment constitutes a niche in which regeneration of new blood vessels and new neurons leads to partial tissue repair after stroke. Cancer metastasis has similar proliferative cellular events in the brain and other organs. Do cancer and CNS tissue repair share similar cellular processes? In this study, we identify a novel role of the regenerative neurovascular niche induced by stroke in promoting brain melanoma metastasis through enhancing cellular interactions with surrounding niche components. Repair-mediated neurovascular signaling induces metastatic cells to express genes crucial to metastasis. Mimicking stroke-like conditions *in vitro* displays an enhancement of metastatic migration potential and allows for the determination of cell-specific signals produced by the regenerative neurovascular niche. Comparative analysis of both *in vitro* and *in vivo* expression profiles reveals a major contribution of endothelial cells in mediating melanoma metastasis. These results point to a previously undiscovered role of the regenerative neurovascular niche in shaping the tumor microenvironment and brain metastatic landscape.

## Introduction

In both health and disease, cellular interactions in the brain occur around a neurovascular niche of neurons, endothelial cells, astrocytes, and stromal cells that maintain blood–brain barrier function and neuronal signaling. Injury to this neurovascular niche during stroke elicits spontaneous but limited repair processes that drastically alter the local environment: creating a cellular niche for tissue repair. Immature neurons (neuroblasts) derived from neural stem cells in the subventricular zone migrate to injured brain regions and differentiate into neurons. These migrating neuroblasts tightly associate with the angiogenic vasculature, which secretes factors essential to post-stroke neurogenesis (Ohab and Carmichael, 2008; Lindvall and Kokaia, 2015). Reactive astrocytes and microglia interact extensively with neuroblasts during post-stroke neurogenesis and engage in signaling via cytokines and growth factors to mediate several parallel cellular functions after stroke, including increased angiogenesis (Ohab and Carmichael, 2008; Lindvall and Kokaia, 2015). Because these cellular elements collectively mediate repair after stroke through angiogenesis, neurogenesis, and tissue reorganization, this microenvironment has been termed the regenerative neurovascular niche (Brumm and Carmichael, 2012).

Brain metastasis presents analogous features with these regenerative neurovascular processes after stroke. In stroke tissue repair, neuroblast migration from a distant brain region into the regenerative neurovascular niche after stroke resembles the migration and localization of a metastatic cancer cell within the brain. Angiogenesis is an essential process in both brain metastasis and repair after stroke (Fukumura et al., 2010; Ergul et al., 2012). Reactive astrocytes interact with both neuroblasts and metastatic cells, and are essential in promoting neurogenesis and metastasis (Klein et al., 2015).

Compelling evidence suggests that similar signaling pathways regulate both stroke repair and brain metastasis (Yao Y. et al., 2016). Tumor suppressor phosphatase tensin homolog (PTEN) is commonly mutated in cancers, and PTEN loss activates oncogenes that reprogram the tumor microenvironment and lead to the formation of brain metastasis. PTEN inhibition after stroke promotes axon regrowth and neuronal repair (Bronisz et al., 2011; Song et al., 2012; Wikman et al., 2012; Zhang et al., 2015). VEGF and angiopoietin signaling mediate endothelial cell proliferation and migration in both cancer metastasis and neural repair after stroke (Hansen et al., 2008; Yang et al., 2010; Ziyad and Iruela-Arispe, 2011; Lange et al., 2016). Matrix metalloproteases (MMPs) also have dual roles in their proteolytic activity, promoting cancer migration and invasion and enhancing angiogenesis after stroke (Lutsenko et al., 2003; Ben-Baruch, 2008; Noel et al., 2008; Izraely et al., 2010; Gialeli et al., 2011; Weitzenfeld and Ben-Baruch, 2013). The TGF-β system regulates cancer cell proliferation in a context-dependent manner (Yang et al., 2008) and mediates tissue reorganization and recovery after stroke (Li et al., 2015). These studies suggest significant overlap in signaling mechanisms of cancer metastasis and tissue repair after stroke.

In short, while the processes of tissue regeneration and metastasis have essentially opposite functional outcomes, the niche components that drive them share similar cellular and molecular features. Both the metastatic tumor and regenerative neurovascular niches involve signaling among a principal cell type (the cancer cell or neuroblast), angiogenic vasculature, reactive astrocytes, and stromal cells. Based on the parallel biology between these two disease processes we asked: (a) could the regenerative neurovascular niche in stroke create a facilitative substrate for brain metastasis, (b) could the synergy between tissue repair processes and metastasis enhance metastatic cell-niche interactions, and (c) does the presence of repair-mediated neurovascular signaling alter the metastatic transcriptome to enhance metastasis?

## Materials and Methods

### Cell Culture

Human brain metastatic melanoma cells YDFR-CB3 were generated in the Witz laboratory as described earlier (Izraely et al., 2012; Klein-Goldberg et al., 2013). YDFR-CB3 cells were cultured in RPMI medium supplemented with 10% heat-inactivated fetal calf serum, 2 mmol/mL L-glutamine, 0.01M HEPES buffered saline, 100 units/mL penicillin, 0.1 mg/mL streptomycin, and 12.5 units/mL nystatin. The YDFR-CB3 cells were transduced with GFP virus (pCDH10-EF1-MCS-2A-copGFP) (Boiko et al., 2010; Quintana et al., 2012). To produce mCherry expressing cells, melanoma cells were transduced with a pQCXIN-mCherry plasmid and selected using 800 μg/mL G418 Sulfate (A.G. Scientific, Inc., San Diego, CA, United States). Immortalized human brain microvascular endothelial cells (hEC) were kindly provided by Dr. Clara Nahmias and Prof. Pierre-Olivier Couraud (Inserm, U1016, Institut Cochin, Paris, France; Cnrs, UMR8104, Paris, France; University Paris Descartes, Paris, France). hEC cells were cultured on collagen type I, rat tail (1:30; BD Biosciences, Bedford, MA, United States) in EBM-2 medium (Clonetics, Cambrex BioScience, Wokingham, United Kingdom) supplemented with 5% heat-inactivated FCS, 2 mmol/mL L-glutamine, 100 units/mL penicillin, 0.1 mg/mL streptomycin, 12.5 units/mL nystatin, 1 ng/mL basic FGF, 10 mM HEPES buffered saline and 1.4 μM hydrocortisone. Human astrocytes (HA; ScienCell Research Laboratories, Carlsbad, CA, United States) were cultured on 0.0015% poly-L-lysine (Sigma-Aldrich, St. Louis, MO, United States) in astrocyte growth medium (AM, ScienCell Research Laboratories) supplemented with 2% heat-inactivated FCS. Cells were routinely cultured in humidified air with 5% CO_2_ at 37°C.

### Modeling Stroke and Metastasis

All animal procedures were carried out in accordance with the National Institutes of Health animal protection guidelines and protocols approved by University of California, Los Angeles Chancellor’s Animal Research Committee. NOD-scid gamma (NSG), a highly immunodeficient mouse model lacking T cells, B cells, and NK cells, was used for these studies (Jackson Laboratories). Inducing strokes in this immunodeficient mouse strain provides a mouse model that allows for the engraftment of human cancer cells without graft rejection and little to no involvement of innate or acquired immunity (Agliano et al., 2008; Chen Q. et al., 2009).

Ischemic strokes were induced in adult male NSG mice aged 2–4 months. Briefly, animals were anesthetized under 2.5–3% isoflurane and positioned on a stereotactic apparatus (Model 940, David Kopf Instruments). To expose the skull, a midline incision was made followed by a transverse incision above the zygomatic arch. A craniotomy was performed to expose the left middle cerebral artery (MCA). The proximal branch of the MCA was cauterized followed by bilateral occlusion of the jugular veins for 15 min. Body temperature was maintained at 37 ± 0.5°C throughout the procedure (RightTemp^TM^, Kent Scientific, Torrington, CT, United States) as measured using a rectal probe. After cauterization, the skin was glued together to cover the skull and animals were returned to their home cages for recovery. Metastasis was modeled by injecting YDFR-CB3^GFP+^ cells (106 cells/50 μl of L15 medium) intracardially 7 days after stroke when the course of regeneration/tissue repair by means of angiogenesis and neurogenesis had reached peak activity (Ohab et al., 2006; Lamanna, 2012). On day 14 after stroke, animals were euthanized and the brains were processed for further experiments.

Sham surgeries were not performed. But surgical incision areas were examined for any tumor homing and no metastasis was observed in these regions.

### Quantitative Analysis of Distribution of Metastasis

Two cohorts consisting of six to eight male NSG mice each were utilized to quantify the distribution of metastasis in different brain regions. One cohort of animals was subjected to stroke and metastasis and the other with metastasis alone. Brains were extracted, fixed in 4% paraformaldehyde and cryosectioned into 100 μm thick sagittal sections while frozen in OCT. Four sections per animal were used to quantify metastasis and the distribution of YDFR-CB3^GFP+^ cells in various brain regions. Sections were imaged, outlined, and GFP^+^ cells were manually counted using a stereoinvestigator (StereoInvestigator MBF Bioscience, Williston, VT, United States) on a Leica DMLB fluorescent microscope (Leica Microsystems, Wetzlar, Germany) with a 40× objective.

### Immunohistochemistry and Confocal Imaging

Immunohistochemistry was performed on free-floating brain sections. Briefly, entire extracted brains were fixed in 4% paraformaldehyde for 24 h and then cryoprotected in 30% sucrose for 48 h. After cryosectioning, 50 μm thick sagittal sections were washed 3× with PBS (phosphate buffered saline) and blocked with 2% horse serum and 5% normal donkey serum in 0.3% Triton. The primary antibodies used are goat anti-DCX (Santa Cruz Biotechnology, CA); rat anti-PECAM (BD Pharminogen); rat anti-GFAP (Invitrogen, IL); Rb anti-albumin (Genetex, CA); mouse anti-MART-1 (Novus biologicals, CO); Rb anti-Tuc-4 (EMD Millipore, CA); mouse antiPSA-NCAM (Millipore, CA); Isolectin Dylight 649 (Vector labs, CA). Sections were incubated with primary antibodies overnight followed by 3× wash with PBS. Secondary antibodies were tested without primary antibodies for non-specific binding were negative (data not shown). Sections were stained with respective secondary antibodies, incubated for 1 h, and washed 3× with PBS. The stained sections were mounted on slides, dehydrated and coverslipped.

For the time course experiments assessing cellular relationships, GFAP, DCX, and lectin immunoreactive areas around the peri-infarct region were imaged. Images were captured of three fields at 100× for astrocytes and 40× for neuroblast and vasculature from three independent sagittal sections of each animal on a confocal microscope (Nikon C2). Z-stacked images of size 512 × 512 were captured. Scanning parameters were maintained constant across groups for consistency.

Human brain metastatic melanoma tissues excised from patients were used to assess neurovascular associations with the metastatic melanoma cells. Paraffin-embedded, slide-mounted human brain sections (5 μm thick) were first immersed in water at 85°C and washed three times, de-paraffinized and hydrated. Double antigen retrieval was performed first using Sodium citrate pH 6 followed by EDTA at pH 8 for 25 min each. As outlined above, immunohistochemistry was performed with MART-1, Tuc-4, and GFAP to identify melanoma cells, neuroblasts and astrocytes, respectively. The Tuc-4, DCX, and GFAP stains were optimized on human hippocampus sections prior to staining the human brain metastatic melanomas.

### Microsphere Injections and Analysis

**FIGURE 1:**
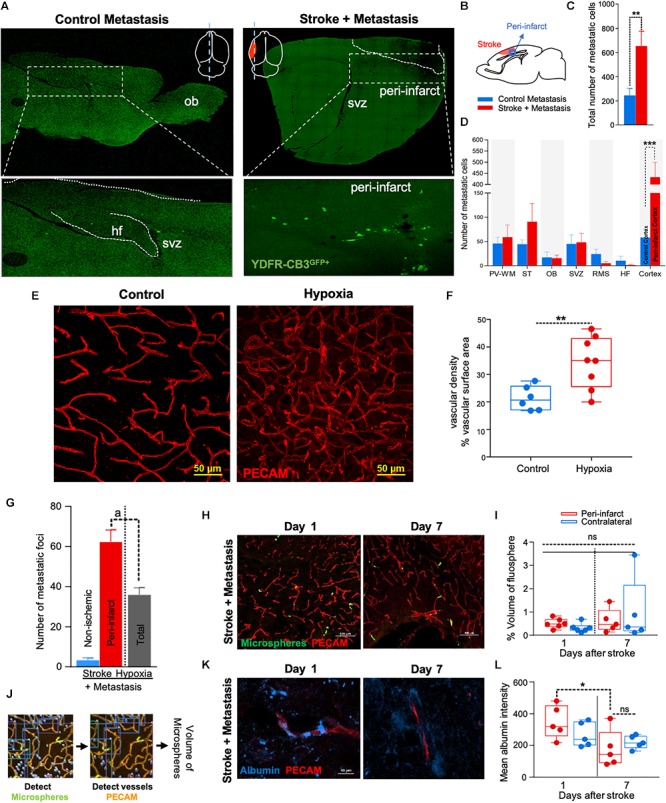
The regenerative neurovascular niche after stroke facilitates brain metastasis. **(A)** Representative brain images from control and stroke groups subjected to melanoma metastasis shown in the top panel. GFP^+^ cells identifies brain metastatic melanoma at high magnification in the bottom panel. **(B)** Schematics showing region from stroke brains and corresponding regions in control quantified. **(C)** Bar graphs of total number of metastatic cells in both control and stroke groups and **(D)** showing quantification and distribution of metastatic cells in different brain regions as mean ± SEM (*n* = 6–7, four sections per animal, ^∗∗^*p* = 0.0082, ^∗∗∗^*p* = 0.0012 Mann–Whitney, two-tailed *t*-test). PV-WM, periventricular white matter; ST, striatum; OB, olfactory bulb; SVZ, subventricular zone; RMS, rostral migratory stream; HF, hippocampus. **(E)** Representative images depicting vascular density in control and hypoxia + metastasis group. **(F)** Box and scatter plots with minimum and maximum percentage vascular density (surface area μm^3^) mean ± SEM (*n* = 6–8), (^∗∗^*p* = 0.0090 unpaired, two-tailed *t*-test) **(G)** Stroke increases. brain metastasis significantly more than hypoxia mediated angiogenesis. Bar graphs showing number of metastatic foci in stroke and hypoxic groups shown as mean ± SEM (*n* = 6–8, four sections/animal, ^a^*p* = 0.003, Mann–Whitney, two-tailed *t*-test). **(H)** Representative images from days 1 and 7 after stroke showing localization of fluorescent microspheres in green and vasculature (PECAM) in red. **(I)** Box and scatter plots with minimum and maximum percentage volume of fluorescent microspheres localized to the peri-infarct and contralateral regions at days 1 and 7 after stroke, mean ± SEM (*n* = 5–6, one-way ANOVA, Holm-Sidak for multiple comparisons *p* = 0.46, ns, non-significant). **(J)** Schematics of the method of quantification of fluorescent microspheres volume localized around the peri-infarct regions. **(K,L)** Representative immunohistochemical images and intensity of endogenous albumin extravasated from days 1 and 7 after stroke and contralateral regions, mean ± SEM (*n* = 5, three regions/animal, ^∗^*p* = 0.0255, one-way ANOVA, Holm-Sidak for multiple comparisons).

Blood–brain barrier leakage and melanoma extravasation were tested with an intracardiac injection of 100 μl of 0.2-μm diameter yellow–green polystyrene fluorescent microspheres (Molecular Probes) which are relatively smaller than metastatic melanoma cells in NSG mice 1 and 7 days after stroke. The microspheres were allowed to circulate for 8 min and the animals were euthanized and brains extracted for immunohistochemistry. 100 μm thick sections were immunostained for the vasculature using PECAM and imaged with Nikon C2 confocal microscope. Intravascular microsphere volume was analyzed (Volocity Improvision 5.5). On the 3D rendered image PECAM positive regions were first detected and selected then regions outside of the PECAM were cropped and the volume of GFP^+^ microspheres accumulated inside these PECAM vessels within the peri-infarct and corresponding contralateral regions was quantified. Schematics are shown in Figure 1J.

### *In vivo* Hypoxia Model

NOD-scid gamma mice were housed for 7 days in a hypoxic chamber (Coy Laboratory Products, Grass Lake, MI, United States) maintained with a continuous flow of 10% O_2_ mixed with N_2_ as background gas. Chamber O_2_ concentration was checked daily and CO_2_ was eliminated by refurbishing the hypoxic chamber with fresh O_2_. Humidity from respiration was controlled with silica desiccant. Animals were provided with food and water *ad libitum* throughout the experiment. After 7 days of hypoxia exposure, YDFR-CB3^GFP+^ cells (10^6^ cells/50 μl of L15 medium) were intracardially injected. The animals were then housed in normoxia/ambient Oxygen levels until day 14. The animals were then euthanized and the brain extracted to analyze the metastatic response to hypoxia-mediated angiogenesis (Harik et al., 1996; Tarnawski et al., 2014).

### Quantitative Analysis of Cellular Associations

Neurovascular associations with melanoma were assessed using DCX-RFP reporter mice. These mice were immunosuppressed with IP injections of Tacrolimus-FK-506 (Cell Signaling, Danvers, MA, United States) beginning with a daily intraperitoneal injection of Tacrolimus (3 mg/kg/day; dissolved in DMSO) 2 days prior to stroke and continuing until the mice were euthanized. The extracted whole brains were fixed, permeabilized, and cryosectioned into 50 μm thick sections. Cell-specific antibodies identified each of these cell types (neuroblasts, astrocytes, and vasculature) through immunohistochemistry.

The cellular association of brain metastatic melanoma with the cells in the regenerative neurovascular niche was assessed by measuring the distances between individual components (astrocytes, neuroblasts) and YDFR-CB3^GFP+^ cells using Imaris software version 8.3.1 (Bitplane^®^). Inflection point analysis and melanoma distance to vascular segments were measured using Volocity Improvision 6. Inflection point measures the number of irregularities within a curvature or change in direction of curvature (Bullitt et al., 2003, 2007). Inflection point ratios were obtained by counting the number of nodes on a vascular segment associated with a metastatic foci and normalizing it to a distant vasculature from the same animal. Vascular segments of the same linear length were compared for every observation for consistency.

### *In vitro* Oxygen Glucose Deprivation (OGD) and Generation of Conditioned Medium

An oxygen-glucose deprivation (OGD) treatment of brain microenvironmental cells was used as an *in vitro* model for stroke (Yang et al., 2012). 5 × 10^5^ human brain endothelial cells and astrocytes were seeded for 24 h. Then, cells were washed with PBSX1 and culture medium was replaced with 0.5% FCS supplemented DMEM medium lacking glucose. The cells were incubated in a hypoxia chamber (Hot box; Billups-Rothenberg Inc, Del Mar, CA, United States) with a gas mixture of: 1% O_2_, 5% CO_2_, and 94% N_2_ for 4 h in 37°C. Cells in control group were treated with 0.5% FCS supplemented DMEM medium with normal glucose levels. These cells were incubated in normoxia for 4 h in 37°C.

Following hypoxia or normoxia, both OGD and control culture media were replaced with a suitable 0.5% FCS supplemented medium (EBM2 for endothelial cells or AM for astrocytes) and incubated in normoxia for 20 h in 37°C, to simulate the reperfusion phase following stroke. Finally, conditioned media from control and OGD cultures was collected, centrifuged and filtered (0.45 μm; Whatman GmbH, Freiburg, Germany) on ice.

### Viability Assay

To examine cell viability of astrocytes and BECs subjected to the OGD treatment, 5 × 10^3^ cells were seeded overnight in coated 96 well-tissue plates. Cells were washed once with PBSX1 and exposed to OGD treatment or normoxia for 4 h with 100 μl relevant DMEM starvation medium. In these studies, AM is astrocyte medium (cat. no. 1801 ScienCell Research Laboratories) and EBM2 is Endothelial Cell Growth Basal Medium-2 (cat. no. 00190860 Lonza). After 4 h the medium was replaced with EBM-2 or AM containing 0.5% serum. Cell viability was determined after 44 h by using Cell Proliferation Kit (XTT, Biological industries, Kibbutz Beit Haemek, Israel), according to the manufacturer’s instructions. Absorbance at 450 nm (OD_450_) was determined for each well using an automated microplate reader (SpectraMax 190; Molecular Devices, Sunnyvale, CA, United States) and subtraction of non-specific readings (measured at 630 nm) was done automatically during the OD_450_ reading.

### Adhesion Assay

Approximately 5 × 10^4^ brain endothelial cells were cultured overnight to form a confluent monolayer, then washed with PBSX1 and subjected to OGD or normoxia for 4 h with 100 μl relevant DMEM starvation medium. After 4 h, the medium was replaced with 100 μl EBM-2 starvation medium containing 0.5% FCS (EC starvation medium) for 20 h.

At the end of the reperfusion phase to model what happens in the *in vivo* setting, brain endothelial cells, *in vitro* were gently washed twice with PBSX1. 1 × 10^5^ m-Cherry labeled melanoma cells diluted in TBSX1 (Tris-buffered saline) containing 2 mM CaCl_2_ were added onto the endothelial monolayer and incubated for 30 min at 37°C to allow adhesion to occur. The total fluorescence signal of labeled cells added to the well before the removal of the non-adherent cells was measured by a fluorescent microplate reader (BioTek FL500, BioTek Instruments, Inc., Winooski, VT, United States) at wavelength of 490/530. Then, the wells were washed three times with PBSX1 to remove non-adherent cells and fluorescence of the adherent cells was measured at wavelength of 490/530. To obtain the percentage of adherent cells, the ratio between the OD of the adherent cells and that of the total cells plated was calculated and multiplied by 100. To determine VCAM-1 involvement, 5 μl/mL VCAM-1 blocking antibody (BD Pharmingen^TM^, San Diego, CA, United States) was added to the m-Cherry labeled melanoma cells that were added onto the endothelial monolayer following the OGD treatment.

### ECM Transmigration Assay

About 5 × 10^4^ brain endothelial cells or astrocytes were cultured overnight. Cells were washed once with PBSX1 and subjected to OGD treatment or normoxia for 4 h with relevant DMEM starvation medium. After 4 h, the medium was replaced with EBM-2 starvation medium for endothelial cells or AM starvation medium containing 0.5% FCS for astrocytes for 20 h. Transwell inserts (6.5 mm diameter polycarbonate membrane with 8.0 μm pores, Corning Costar Corp., New York, NY, United States) were coated with 100 μg/mL collagen type I for 1 h at 37°C. After two washes with 200 μl PBSX1, 1 × 10^5^ melanoma cells were added to the Transwell for 1.5 h at 37°C, and the cells were allowed to settle on the semipermeable membrane of the Transwell. The Transwells were placed in the well plates containing the OGD or control endothelial cells or astrocytes and melanoma cells were allowed to transmigrate through the ECM (Collagen) and membrane overnight toward the lower chamber.

At the end of the migration period, melanoma cells in the upper chamber that had not transmigrated were removed using cotton swabs, followed by a gentle wash of the bottom side of the Transwell inserts in PBSX1 and fixation in absolute methanol for 5 min. The Transwell inserts were washed in PBSX1 and stained with Diff-Quik^®^ staining kit (Medion Diagnostics, Düdingen, Switzerland).

Transwells were photographed using Olympus IX53 inverted microscope fitted with an Olympus DP73 camera Olympus Inc., Hamburg, Germany), two fields per Transwell in duplicate Transwells. Cells per field were counted or analyzed using ImageJ software.

### Cytokine Array

The supernatants from OGD and control astrocytes and endothelial cells were analyzed for the secretion of 102 cytokines, using the Proteome Profiler Human XL Cytokine Array Kit (R&D systems, Inc., Minneapolis, MA, United States), according to the manufacturer’s instructions. A minimum of a 20% difference in pixel density between OGD and control samples was accounted as a change in secretion level.

### Tissue Processing and FACS

Male NSG mice aged 2–4 months were subjected to stroke followed by metastasis at day 7 as previously described. To eliminate any confounding factors and since estrogen is neuroprotective, male animals were used for this study. On day 14 after stroke, the animals were anesthetized with isoflurane, decapitated, and the peri-infarct areas were carefully dissected using a sterile lancet. Corresponding areas dissected from the non-stroke side served as a control. The dissected tissue was further cut into fine bits with a sterile blade, triturated with a pipette tip, and digested in 3 mL of collagenase (3 mg/mL, Roche Diagnostics) at 37°C for 25 min at 170 rpm. Hibernate buffer without calcium (Brainbits, Springfield, IL, United States) was used to maintain tissue viability and pH during the process of dissection and digestion. The digestate was triturated and washed twice at 3,000 rpm for 10 min with this hibernate medium. To enrich the human population and deplete potential mouse cells from the neural tissue, the pellet was re-suspended in low fluorescence hibernate media and the MACS- mouse cell depletion kit (Miltenyi Biotec, San Diego, CA, United States) was used as per manufacturer’s instructions. Brain metastatic melanoma cells were negatively selected through immunomagnetic separation as provided by the manufacturer’s protocol.

The enriched cell suspensions were stained with melanoma marker MCSP conjugated with PE (Human; MCSP-PE, 10 μL antibody/107 cells, Miltenyi Biotec, San Diego, CA, United States) by incubation at 4°C for 10 min. The cells were washed with 2 mL of Hibernate medium, re-suspended in low fluorescence hibernate medium, and maintained on ice during FACS isolation. Cell were co-stained with DAPI to sort for live cells. DAPI, PE, and GFP gates were set using positive and negative controls prior to sorting melanoma cells. Metastatic melanoma cells were collected via FACS (FACsARIA, Becton Dickinson, UCLA FACS Core) into low fluorescence medium.

### RNA Isolation (Both *in vivo* and *in vitro*)

FACS-sorted cells were pooled from six to eight animals and the RNA was extracted using an RNA-Microprep kit (Zymo-Research) and eluted into 12 μL RNAse-free H_2_O. RNA-quality was verified on an Agilent Bioanalyzer using an Agilent picokit with relative concentrations that ranged from 170 to 1,733 pg/μL. Samples with RIN > 6 were sent for sequencing. For the extraction of RNA from *in vitro* melanoma cultures, YDFR-CB3 cells were treated with conditioned media of either OGD or normoxic conditions on hEC/h-Astro. Melanoma cells were then washed twice with PBSX1 and RNA was extracted using the NucleoSpin miRNA kit (Macherey-Nagel, Düren, Germany) according to the manufacturer’s instructions.

### RNA Sequencing and Transcriptome Analysis

RNA sequencing was done by the UCLA Neuroscience Genomics Core at UCLA. Briefly, for *in vivo* samples, library was prepared using Ovation RNA Ultra Low Input (500 pg) kit (Nugen manufacturer) and TruSeq Nano DNA library preparation kit (Illumina manufacturer). Amplified cDNA was purified and validated followed by library preparation. Preparation included 69 bp paired end reads. Samples were sequenced by Hi-Seq 2500 over 7 RapidRun lanes, corresponding to about 1.8 samples per lane (80 million reads per sample) for the *in vivo* datasets. *In vitro* samples were sequenced over 12 RapidRun lanes, corresponding to about 2.3 samples per lane (65 million reads per sample).

RNA sequencing performed on melanoma cells conditioned with either astrocyte/endothelial (OGD/control) conditioned media resulted in 60,906,978 to 25,890,188 with 40.18 to 75.37% of uniquely mapped reads. Samples with a minimum of 10 transcript counts were used and κ = 3 for *in vitro* batch correction was applied in edgeR package. Melanoma cells exposed to endothelial conditioned media with or without OGD resulted in 4717 significant DE (differentially expressed) genes (*p* < 0.05). Further filtering for log_2_ (fold change) ≥±0.5 resulted in 3,073 genes of which 712 were upregulated and 2,361 were downregulated. Melanoma cells exposed to astrocyte conditioned media with or without OGD resulted in 1,127 significant DE genes (*p* < 0.05). Further filtering for log_2_ (fold change) ≥ ±0.5 resulted in 362 genes of these, 252 were upregulated and 111 were downregulated.

### Read Mapping, Assembly, and Expression Analysis

Short reads were aligned using STAR to the latest human reference genome (GRCh38). Fragment counts were determined by HTseq program using RefSeq gene and Ensembl transcript model (Conesa et al., 2016).

### Differentially Expressed (DE) Gene Analysis Between *in vivo* and *in vitro* Datasets

Due to the large number of samples, unexpected batch effects were expected. Removal of unwanted variation (RUV) is effective to remove such batch effect. For *in vitro* datasets, raw counts were filtered (minimum 10 counts for at least eight samples), and then normalized by TMM followed by RUV correction with *k* = 3 (Risso et al., 2014). Differentially expressed genes were determined using edgeR. For *in vivo* samples, differential expression analysis was done without RUV correction (Robinson et al., 2010).

### GSEA (Gene Set Enrichment Analysis)

Within this analysis, a hallmark gene set is defined by a collection that summarizes specific well-defined biological states, processes, or diseases with coherent expression changes in many molecules within a system (Subramanian et al., 2005; Liberzon et al., 2015). A unique Stroke-MET transcriptome was identified by subtracting metastatic melanoma gene expression in non-stroke vs. metastatic melanoma gene expression in the stroke side. The set of differentially regulated genes in the Stroke-MET transcriptome were probed for common regulatory elements, which might be controlling gene expression. These are termed upstream regulators, as they are predicted to regulate genes that are significantly induced or downregulated in the Stroke-MET transcriptome. These upstream regulators were tested for significance based on an enrichment score (Fisher’s exact test *p*-value overlap of predicted and observed regulated gene sets) and activation *Z*-scores (to test for their association with up or down-regulation of their associated genes) in the Stroke-MET transcriptome.

### Network Generation and Properties

To explore the molecular interactions, transcription profiles that overlap between *in vivo* and *in vitro* datasets were analyzed. Overlapping molecular transcription profiles between *in vivo* and *in vitro* datasets with high focus numbers were analyzed for network properties. Network properties such as degree and betweenness centralities were assessed with CytoNCA (Tang et al., 2015).

### SPIA (Single Primer Isothermal Amplification)

To further validate candidate gene expression profiles obtained from the RNAseq datasets, metastatic melanoma cells were FACS isolated as described above from independent experimental samples. The resultant RNA were in pg/μl range and this was amplified using the Nugen Ovation^®^ Pico WTA System V2. RNA amplification was performed based on the protocol and instructions provided in the kit using the Ribo-SPIA technology.

### qPCR Analysis of Molecules Associated With Both the Metastatic Microenvironment

The SPIA amplified RNA were used to test the gene expression fold change between the metastatic melanoma cells associated with the stroke and non-stroke sides of the brain. 1.25 ng/μl of cDNA was generated from each sample and used to detect expression using Roche Light Cycler 480 SYBR Green I Master Kit. RT-qPCRs were performed using custom primers and human-GAPDH as the house keeping gene.

### CLARITY (Clear Lipid-Exchanged Acrylamide-Hybridized Rigid Imaging Compatible Tissue Hydrogel)

Brains exposed to both stroke and metastasis in DCX-RFP mice were extracted. Brains were left intact and were optically cleared using a CLARITY-PACT protocol modified for passive and quick clearing (Chung and Deisseroth, 2013; Tomer et al., 2014; Yang et al., 2014). Briefly, each DCX-RFP animals subjected to stroke and metastasis were perfused with 100 μl of vascular lectin (Vector Laboratories) prior to euthanization. The brains were then fixed with 4% paraformaldehyde and incubated at 40°C with 30 mL of cold hydrogel for 5 days. Prior to polymerization of the hydrogel, the tubes were vacuum sealed, purged with nitrogen gas and immediately sealed. The tubes were then incubated at 370°C for 4–5 h to allow for adequate polymerization of the hydrogel bound tissue. After polymerization, excess gel was carefully removed and the whole brain was cut into 2 mm thick sagittal sections using a brain mold. The brains were passively cleared with a solution of 200 mM boric acid, 8% SDS, pH 8.5 at 400°C. Clearing solution was refreshed every day until the sections were transparent. The tissue sections were rinsed with PBS and mounted in Focus Clear (CelExplorer Labs, Hsinchu, Taiwan) solution. The sections were imaged under a confocal microscope (Nikon C2) at 10× and the stacked images were constructed into video files.

### Statistical Analysis

Data analysis was done with Microsoft Excel and GraphPad Prism version 7.00 (GraphPad Software, La Jolla, CA, United States). Statistical significance was determined by two-tailed unpaired Student’s *t*-test when comparing two groups, one-way ANOVA, or two-way ANOVA wherever appropriate for experiments consisting of more than two groups. Disease and time course interaction and effects were done followed with *post hoc* test with wither Holm-Sidak or Fisher’s least significant difference (LSD) and are specified on each figure legends. Significance values are presented as mean ± standard error of the mean (SEM) for the *in vivo* experiments and mean ± SD for the *in vitro* experiments.

## Results

### The Regenerative Neurovascular Niche Enhances Melanoma Brain Metastasis

NOD-scid gamma mice were subjected to distal middle cerebral artery occlusion (MCAo), a common, clinically relevant stroke model (Agliano et al., 2008; Chen B. et al., 2009), and injected with YDFR-CB3, a melanoma line selected for its ability to form brain macrometastases (Izraely et al., 2010). This is an ideal model to test the preferential localization of a metastatic tumor cell, since melanoma develops from the neural crest and commonly metastasizes to brain (Boiko et al., 2010; Izraely et al., 2012; Quintana et al., 2012; Marzese et al., 2015). YDFR-CB3^GFP+^ were administered via intracardiac injection 7 days post-stroke, when angiogenesis and neurogenesis peak and the regenerative neurovascular niche is most active (Morris et al., 2003; Ohab et al., 2006; Lamanna, 2012). The total number of metastatic cells in the brain was significantly greater in the stroke group compared to controls 7 days post-injection (*p* = 0.0082, control metastasis 245 ± 57, stroke + metastasis 653 ± 122) (Figure 1A–C and Supplementary Figure 1). The YDFR-CB3^GFP+^ cell distribution was quantified in the peri-infarct, where angiogenic vessels are predominantly found, and in other brain regions in which angiogenesis or tissue repair is absent (Cavaglia et al., 2001): periventricular white matter, striatum, hippocampus, and olfactory bulb. On average, 433 ± 67 YDFR-CB3^GFP+^ cells preferentially localized to the peri-infarct compared to other regions in stroke and 73 ± 23 cells were found in corresponding cortical regions in control metastasis (*p* = 0.0012, *n* = 6–7) (Figure 1D). This data indicates that metastatic melanoma preferentially localizes to the regenerative neurovascular niche.

### Metastasis to the Regenerative Neurovascular Niche Occurs Independently of Hypoxia Mediated Angiogenesis

Within the regenerative neurovascular niche after stroke, there are several potential cellular signaling sources that might influence brain metastasis. Angiogenic endothelial cells may directly initiate melanoma cell localization and establish metastasis. Instead, melanoma cells may localize to the regenerative multicellular environment of endothelial cells, astrocytes and neuroblasts within the peri-infarct cortex, the combination of these cells constituting a niche in which cell–cell communication is the interaction of the cells (the niche) rather than that of a single cell (Carmichael, 2016). To test this, we used a model of global angiogenesis that does not produce the regenerative neurovascular niche or neurogenesis seen in stroke. Chronic hypobaric oxygen mediates angiogenesis by globally increasing cerebral vascular density (Figure 1E,F) (Harik et al., 1996; LaManna et al., 1998). If angiogenesis itself is inducing melanoma cell localization, this generalized angiogenic state would be expected to result in the preferential localization of vascular melanoma cells. A cohort of hypoxic animals subjected to 10% hypoxia for 7 days was injected with metastatic melanoma cells intracardially and the number of metastatic foci was assessed 7 days after injection. Each metastatic focus represents an incidence of metastasis and is defined by a cluster of melanoma cells that has localized and developed into a colony. The 62 ± 6 metastatic foci observed in the peri-infarct region was significantly higher than 36 ± 4 of the hypoxic cohort (*p* = 0.003, *n* = 6–8) (Figure 1G). This suggests that the regenerative response after stroke significantly enhances brain metastasis localization of melanoma compared to hypoxia-mediated angiogenesis alone.

### Metastasis to the Regenerative Neurovascular Niche Occurs Independently of Vascular Hemodynamic Changes

Stroke alters blood flow dynamics and blood–brain barrier permeability in the peri-infarct tissue (Chen Q. et al., 2009) into which metastatic melanoma preferentially localize. To investigate whether this preferential localization of melanoma is simply due to the alterations in blood flow or vascular permeability changes induced by stroke, we injected two cohorts of animals with fluorescent microspheres (0.2 μm diameter) at 1 and 7 days after stroke. These microspheres lodge in the capillary bed and allow for tracking and quantification of differences in blood flow to different brain regions (Springer et al., 2000). The volume of microspheres accumulated in angiogenic vessels within the peri-infarct and corresponding contralateral regions was quantified. No significant difference was observed between microsphere accumulation in the ischemic and non-ischemic hemispheres at days 1 and 7 after stroke (Figure 1H–J). This suggests that blood flow to the peri-infarct is not significantly altered at these time periods after stroke and that blood flow changes are not the cause of the observed preferential melanoma cell metastasis to this region.

Vascular leakage due to blood–brain barrier breakdown leads to extravasation of plasma proteins (Abraham et al., 2002; Tenreiro et al., 2016). To determine if blood–brain barrier permeability changes could contribute to preferential localization of melanoma, we stained brain sections for endogenous albumin extravasated at days 1 and 7 after stroke (Abraham et al., 2002). The mean intensity of extravascular albumin was elevated at day 1, but significantly reduced at day 7 and was not different from the corresponding contralateral region at day 7 (*p* = 0.0255, *n* = 5) (Figure 1K,L). These findings support the idea that the localization of metastatic melanoma cells to the peri-infarct at day 7 are not due to vascular hemodynamic changes induced by stroke, and may be due to specific cellular or molecular features of these vessels within the angiogenic neurovascular niche.

### Neuroblast-Associated Vessels Within the Regenerative Neurovascular Niche Mediate Brain Metastasis

**FIGURE 2:**
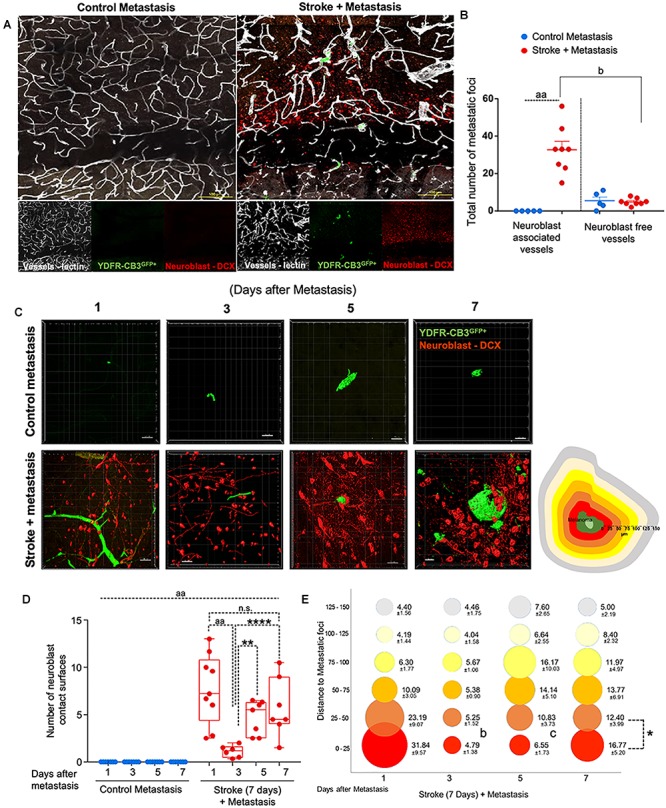
Repair augments direct cellular interactions between the metastatic cell and neurovascular niche. **(A)** Immunohistochemical analysis of metastatic melanoma cells shown as GFP^+^ green, lectin-labeled vasculature in white and DCX^+^ neuroblasts in red. **(B)** Quantitative analysis of metastatic cell interaction with vessels associated with or without neuroblasts shown as scatter plot with mean ± SEM (*n* = 5–8, four sections/animal, ^aa^*p* < 0.0001; ^b^*p* = 0.0354, Kruskal–Wallis, one-way ANOVA, Dunn’s for multiple comparisons). **(C)** Representative brain images of metastatic foci (GFP^+^) and neuroblasts (RFP^+^) over time (left). Control groups did not associate with neuroblasts in the corresponding brain region. Schematic representation of distance bins (25 μm intervals) used to analyze neuroblast distribution around metastatic foci. Bin closest to melanoma is shown in bright red and increasing distances showing in gradients of yellow and the farthest depicted in gray (right). **(D)** Box and scatter plot showing number of neuroblast contact surfaces interacting with metastatic foci. Mean ± SEM (*n* = 6–9, ^∗∗^*p* = 0.0092, ^∗∗∗∗^*p* = 0.0006, ^aa^*p* < 0.0001; one-way ANOVA, Holm-Sidak *post hoc* test used for multiple comparisons). **(E)** Bubble plot depicting density and distribution of neuroblasts around metastatic foci at 25 μm distances Mean ± SEM (*n* = 5–8, ^b^*p* = 0.033, ^c^*p* = 0.033, ^∗^*p* < 0.0164; one-way ANOVA, Holm-Sidak *post hoc* test used for multiple comparisons). Bubble size represents number of neuroblasts and increasing distances are color coded as shown in schematic.

**FIGURE 3:**
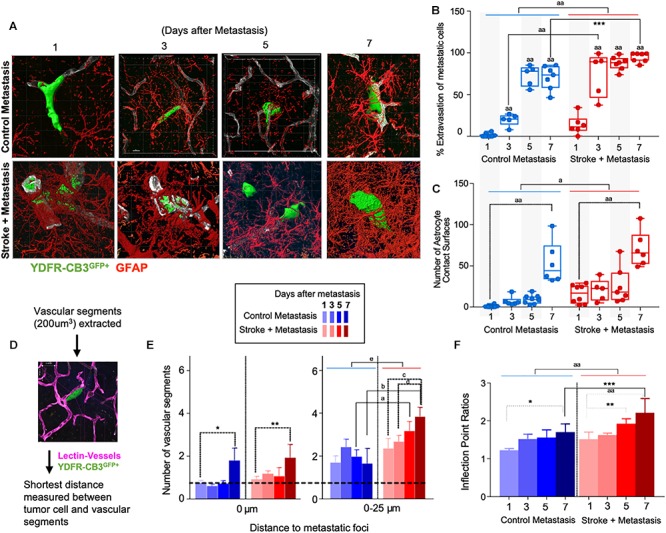
Repair enhances direct cellular interactions between the metastatic cell and astrocytes and increases neovascularization. **(A)** Representative images showing metastatic melanoma interaction with reactive astrocytes during melanoma extravasation. **(B)** Quantitative analysis of % volume of metastatic cell extravasated. Mean ± SEM [^∗∗∗^*p* = 0.0082, ^aa^*p* < 0.0001, two-way ANOVA, Holm-Sidak *post hoc* test used for multiple comparisons (*n* = 5–8)]. **(C)** Astrocyte coverage at corresponding time points around each metastatic foci. Mean ± SEM [^aa^*p* < 0.0001, ^a^*p* = 0.0006, two-way ANOVA, Holm-Sidak *post hoc* test used for multiple comparisons (*n* = 5–8)]. **(D)** Methodology of analysis of neovascularization and infection point analysis. **(E)** Changes in neovascularization response over time mean ± SEM, (*n* = 5–8), (^∗^*p* = 0.0424, ^∗∗^*p* = 0.0349, ^a^*p* = 0.0454, ^b^*p* = 0.0015, ^c^*p* = 0.0163, ^d^*p* = 0.0474, ^e^*p* = 0.0009, two-way ANOVA, Fisher LSD). **(F)** Bar graphs showing temporal changes in inflection point ratios [^∗^*p* = 0.0032, ^∗∗^*p* = 0.0215; ^∗∗∗^*p* = 0.0012; ^aa^*p* < 0.0001; two-way ANOVA, Holm-Sidak *post hoc* test used for multiple comparisons, (*n* = 5–8)].

Intercellular communication between cancer cells and host tissue is critical for metastatic localization and tumor progression (Termini et al., 2014). After stroke, neural stem cells in the subventricular zone divide and differentiate into immature neurons (termed neuroblasts), which migrate to brain tissue adjacent to the stroke site and closely associate with angiogenic blood vessels (Cook et al., 2017). We assessed the influence of neuroblasts on metastatic melanoma localization to angiogenic vessels within the regenerative neurovascular niche using genetic labeling: a DCX-RFP mouse, in which red fluorescent protein is expressed in neuroblasts and serves as an accurate reporter of post-stroke neurogenesis (Cook et al., 2017). In these studies, vessels are labeled with perfused lectin, and are rendered in white for visualization in the black photomicrograph background (Figure 2, 3).

Within 1 day after metastasis, 33 ± 5 metastatic foci seeded in neuroblast-associated vessels in the peri-infarct of stroke animals (*p* = 0.0354, *n* = 5–8), while there was no such metastatic localization in the corresponding control cortex (*p* < 0.0001, *n* = 5–8) (Figure 2A,B). On average, five metastatic foci localized to vessels without neuroblasts in both control and stroke mice groups. This suggests that blood vessels that support post-stroke neurogenesis govern the preferential localization of metastatic melanoma in the regenerative neurovascular niche.

### Tissue Repair Augments Direct Cellular Interactions Between Metastatic Cells and Neurovascular Niche Components

As metastatic melanoma cells localize to the regenerative neurovascular niche, they associate with distinct cellular niche elements over time, such as angiogenic vessels, astrocytes, and neuroblasts (Termini et al., 2014). These associations between metastatic cells and niche components at day 7 after metastasis are shown on CLARITY-processed tissue after stroke (Supplementary Video 1). To further determine cellular relationships between melanoma cells and this niche, we examined spatiotemporal interactions between the metastatic cells and neurovascular components at 1, 3, 5, and 7 days after metastasis in control and stroke cohorts. These time points reflect early events in metastasis that determine metastatic cell survival and extravasation.

### Metastatic Melanoma Interact With Neuroblasts Within the Regenerative Neurovascular Niche

To examine direct interactions between metastatic cells and neuroblasts (the two principal cells of the metastatic and regenerative neurovascular niches) the number of neuroblasts-melanoma contact surfaces were quantified over time in the peri-infarct and corresponding control cortex at 1, 3, 5, and 7 days after metastasis. On average, 7.7 ± 1.2 contact surfaces from neuroblasts interacted with the metastatic foci at day 1. Following this, the number of interactions significantly reduced at day 3 to 1.1 ± 0.3 contact surfaces (*p* < 0.0001, *n* = 6–9). Further, at days 5 and 7, neuroblast contact surfaces steadily increased compared to day 3 (4.7 ± 0.7, *p* = 0.0092, and 5.7 ± 1.2, ^∗∗∗∗^*p* = 0.0006, respectively) in the stroke cohort. No interactions were found in the corresponding control regions due to the absence of neuroblasts in analyzed control regions (Figure 2C,D).

To determine the spatiotemporal distribution of neuroblasts around melanoma cells, we analyzed neuroblast counts at 25 μm distance intervals from the metastatic foci periphery (Figure 2E). In the stroke cohort, 32 ± 10 neuroblasts were found at 1 day after metastasis within a proximity of 25 μm. At day 3, 5 ± 1 neuroblasts surround metastatic foci in equal distributions at increasing distances. At days 5 and 7, more neuroblasts migrate closer to the metastatic foci, with 7 ± 2 neuroblasts surrounding metastatic cells at day 5 and 17 ± 5 neuroblasts at day 7 (*p* = 0.0164, *n* = 5–8).

This data suggests that neuroblasts around the metastatic foci associate with melanoma in two phases. At day 1, there is a vascular phase: melanoma cells form a large number of contacts with neuroblasts as they associate with neuroblast-associated vasculature in the peri-infarct (Figure 2A,B). After melanoma extravasation at day 3, melanoma separate from the neuroblasts in the niche, followed by a tissue phase: melanoma cell associations with neuroblasts increase until day 7 in the regenerative neurovascular niche (Figure 2C–E). These distinct phases in neuroblast-metastasis interaction are unique to the regenerative neurovascular niche. The corresponding cortical regions in the metastasis-only (no stroke) group had no metastatic foci associated with neuroblasts.

### Tissue Repair After Stroke Enhances Association Between Extravasated Metastatic Cells and Surrounding Reactive Astrocytes

Since reactive astrocytes play a crucial role in the neurovascular niche and in brain metastasis (Klein et al., 2015), we assessed if the regenerative neurovascular niche enhances reactive astrocyte contact surfaces with the metastatic melanoma at 1, 3, 5, and 7 days after stroke. Extravasation of metastatic cells initiates after 3 days in controls and within 1 day in stroke group. A two-way ANOVA indicated that this accelerated time course of extravasation was temporally dependent upon the presence of regenerative neurovascular niche (*p* = 0.0008, variance 4.33%) (Figure 3A–C). Corresponding to the extravasation, astrocytic processes begin interacting with extravasated melanoma early at day 1 in the stroke group compared to day 3 in controls. Total astrocytic contacts increase from 7 ± 2 at 3 days to 9 ± 2 at 5 days to 53 ± 10 at 7 days in control metastasis (*p* < 0.0001, *n* = 5–8) while they increase from 20 ± 6 at day 3 to 26 ± 8 at day 5 to 70 ± 9 at day 7 in stroke+metastasis (*p* < 0.0001, *n* = 5–8). Astrocyte contact surfaces linearly increase as metastatic cells extravasate in both control and stroke (pooled slope: 7.706). The presence of a regenerative neurovascular niche significantly enhanced melanoma–astrocyte interactions independent of time (two-way AVOVA, *p* = 0.0006) (Figure 3).

### Regenerative Vasculature Is More Susceptible to Metastatic Cell-Induced Remodeling

The progression of brain metastasis is dependent upon the hijacking or recruitment of adjacent vessels, a process known as vascular cooption (Qian et al., 2016). To examine the temporal increase in vessel recruitment with metastatic cells, the distance between individual vessel segments and metastatic cells were measured. After metastasis, direct vessel contact with metastatic cells significantly increased from day 1 to day 7 in both control and stroke cohorts with 0.75 ± 0.12 vessels in contact with metastatic melanoma at day 1 and 1.8 ± 0.58 at day 7 in controls (*p* = 0.0424, *n* = 5–8), and 0.93 ± 0.13 at day 1 and 1.93 ± 0.62 at day 7 in the stroke cohort (*p* = 0.0349, *n* = 5–8). While no difference in direct vessel associations was observed between cohorts, there was a significant increase in close proximity vessels in the stroke groups compared to controls. Within a periphery of 25 μm from the metastatic foci, vessel association significantly increased from day 1 to day 7 in the stroke groups compared to controls, with 1.65 ± 0.71 vessels at day 7 in control vs. 3.85 ± 0.43 at day 7 (*p* = 0.0015, *n* = 5–8) in stroke (Figure 3D,E). A two-way ANOVA indicated that the regenerative neurovascular niche significantly influenced vascular associations independent of temporal effects (*p* = 0.0009, variance 19.71%). This data suggests that vascular cooption is essential to metastasis as early as 7 days and that this vascular remodeling is significantly augmented in the regenerative neurovascular niche.

When metastatic cells’ co-opt or route along vasculature, they induce local irregularities in vessels (Bullitt et al., 2003, 2005). To assess these vessel irregularities, we utilized inflection points, which measure the number of irregularities or changes in direction within a curvature. We measured inflection points on vessels associated with metastatic cells and normalized over inflection points on vasculature not associated with metastatic cells. Metastasis alone significantly increased inflection points on associated vasculature at day 7 compared to day 1 (*p* = 0.0032, *n* = 5–7). However, after stroke, inflection point ratios progressively increased at day 7 compared to day 1 (*p* < 0.0001, *n* = 5–7) and were significantly higher compared to controls at day 7 (*p* = 0.0012, *n* = 5–7) (Figure 3F). A two-way ANOVA indicated that stroke significantly enhanced inflection points independent of time. This data suggests that metastasis enhances vascular irregularities as early as 7 days, and angiogenic vessels within the neurovascular niche not only preferentially localize melanoma cells but are then more susceptible to melanoma-induced vascular remodeling.

### Human Brain Metastatic Melanoma Interact With Neuroblasts and Astrocytes

**FIGURE 4:**
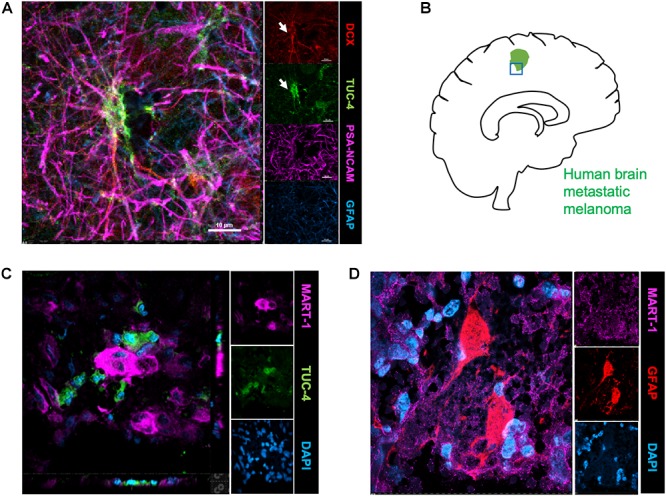
Human brain metastatic melanoma interact with neuroblasts and astrocytes. **(A)** Representative image depicting characterization of antibodies on human brain samples. Human hippocampus section showing staining for DCX, PSA-NCAM, GFAP, and Tuc-4. DCX positive neuroblasts colocalize with Tuc-4. **(B)** Schematics showing human brain tumor margin immunostained for neuroblasts and astrocyte association. **(C)** Immunohistochemical representative images of brain metastatic melanoma show novel interactions with neuroblasts excised from human patients (4/15 show close neuroblast–melanoma interactions). **(D)** Representative images of brain metastatic melanoma from human patients show interactions with astrocytes.

To identify the possible interactions of neuroblasts, astrocytes, and metastatic cells in human brain tumors, we immunostained representative human brain metastatic melanomas FFPE sections obtained from elective surgeries with biomarkers for neuroblasts (Tuc-4), astrocytes (GFAP) and melanoma (MART-1). Fifteen distinct patient samples of brain metastasis were immunostained and 4 of 15 tumors showed co-localization of metastatic with neuroblasts (Figure 4). The presence of interactions between human melanoma and surrounding neural cells implicated in stroke repair suggests that elements of the regenerative neurovascular niche play an important role in mediating melanoma metastasis.

### Isolation and Sequencing of Stroke Responsive Metastatic Melanoma Cells

**FIGURE 5:**
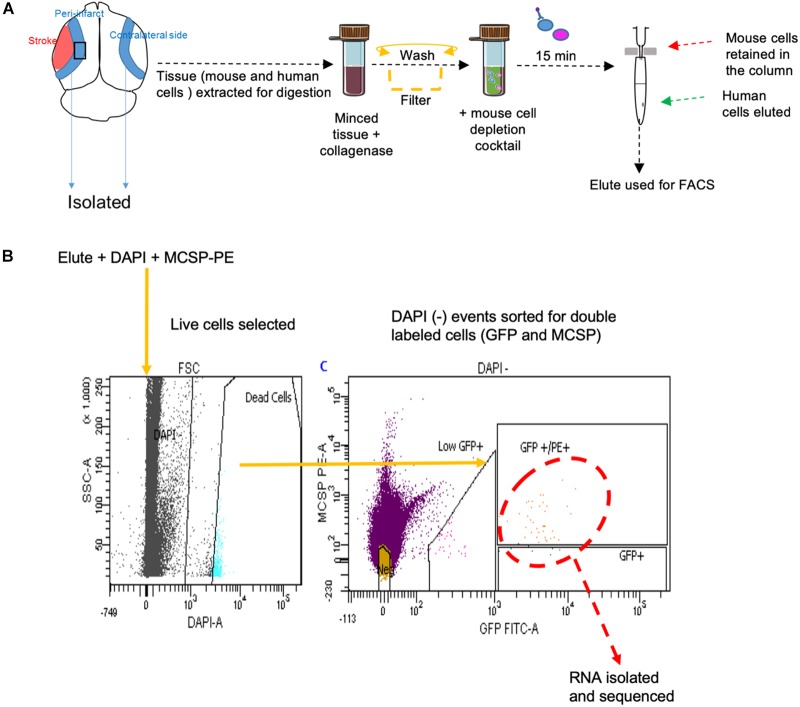
**(A)** Schematic representation of tissue processing and FACS sort methodology performed. **(B)** Gating areas used during FACS and the GFP and MCSP-PE double positive cells were isolated for RNA sequencing. MCSP, melanoma-associated chondroitin sulfate proteoglycan.

FACS sort was performed on YDFR-CB3^GFP+^ cells isolated from both the peri-infarct region and the corresponding non-stroke side. We developed an efficient method to purify and enrich these metastatic cells using magnetic bead columns that negatively selected human cells in the elute and retained the mouse cells in columns bound to mouse cell specific antibodies conjugated to beads. This elute enriched for human cell population was gated for GFP and MCSP-PE channels in FACS. For each sample, metastatic cells isolated from two to three mice were pooled from the peri-infarct regions and six to eight animals were pooled from the contralateral side (*n* = 5–7) (Figure 5).

RNA sequencing performed on the metastatic melanoma cells isolated *in vivo* from the stroke and the non-stroke side of the brain resulted in 74–101.5 million reads per transcriptome. The percent of uniquely mapped reads ranged from 79.37 to 86.23%. Samples with a minimum of 10 transcript counts were used and batch correction was applied to remove unwanted variation with *k* = 2 (Risso et al., 2014). Differentially expressed genes between the metastatic cells isolated from the peri-infarct region (stroke side) and the corresponding non-stroke brain regions (contralateral hemisphere) were determined with the bioconductor edgeR package (Robinson et al., 2010).

### Molecular Signaling Systems in Regeneration-Potentiated Brain Metastasis

**FIGURE 6:**
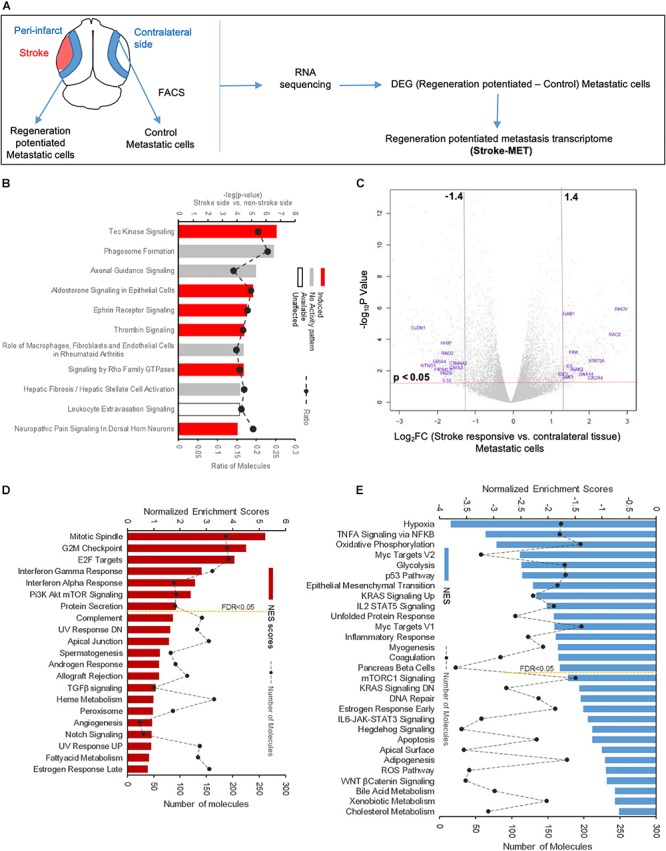
Molecular signaling systems in regeneration-potentiated brain metastasis. **(A)** Schematic representation of flow of transcription profiling and regenerative neurovascular niche components interacting with metastatic cells. Significant and differentially expressed genes between regeneration-potentiated and contralateral metastatic cells as determined by log_2_ fold change > 0.5 and *p* < 0.05 (Stroke-MET). **(B)** Top differentially regulated canonical pathways with largest negative logP shown in metastatic melanoma cells from the regenerative neurovascular niche compared to contralateral tissue. The dashed line represents the ratio of the number of molecules in the dataset over the number of molecules in the pathway. **(C)** Volcano plot showing *p*-values correlated with log_2_ fold change in metastatic cells isolated from regenerative neurovascular niche vs. contralateral side. Genes systematically identified from top pathways from **(B)**, are marked in blue. **(D,E)** GSEA of hallmark genes up and downregulated in the Stroke-MET transcriptome. Estimated probability of FDR < 25% represent 3/4 chances of a gene set being valid.

To determine the unique gene expression changes that occur within metastatic melanoma in the presence of regenerative neurovascular signaling, we sequenced total RNA extracted from FACS-isolated, regeneration-potentiated metastatic melanoma cells *in vivo* from the peri-infarct cortex vs. metastatic melanoma cells not localized to the neurovascular niche after stroke. Cell-specific RNA-sequencing identified 4,083 differentially expressed genes between metastatic melanoma cells (stroke vs. non-stroke *p* ≤ 0.05). Filtering this significantly regulated gene list for log_2_ (fold change) ≥±0.5 resulted in 2,264 genes in regeneration-potentiated metastatic melanoma cells vs. brain metastases not in the stroke site. Because this transcriptome comes specifically from a subtraction of metastatic melanoma gene expression in non-stroke vs. the potentiated state of stroke, this is a unique transcriptome of melanoma specific to the regenerative neurovascular niche, termed the Stroke-MET transcriptome: 1,118 genes are upregulated and 1,146 are downregulated in metastatic melanoma within the regenerative neurovascular niche compared to melanoma brain metastases outside of this niche. The organization of differentially regulated genes in the Stroke-MET transcriptome was studied by association into top-scoring canonical molecular pathways (Figure 6). Canonical pathways strongly activated in the Stroke-MET transcriptome include axon guidance, ephrin receptor signaling, phagosome formation, and thrombin signaling. A volcano plot of the significant differentially expressed genes identifies those genes with the highest fold change and most significant expression in the Stroke-MET transcriptome (Figure 6). Twenty-two of these genes are reported to have extensive roles in determining metastasis as tabulated in Table 1.

**Table 1:** Functionally relevant gene identified from Stroke-MET transcriptome.

Gene	Log_2_ FC	Pathways regulated (Figure 6)	Reported function(s) (database used: pubmed)	References
NTNG1	-1.922	Axon guidance	Axonal growth protein also expressed in stroma of human PDAC.	Rollins, 2016
			High methylation status associated with poor outcomes in colorectal cancer.	Sho et al., 2017
			Promote axon outgrowth.	Lin et al., 2003
			Unreported in brain metastasis	
HHIP	-1.775	Axon guidance	Regulates endothelial cell proliferation/migration through Hedgehog pathway inhibition	Yan et al., 2014
			Gli-Hedgehog signaling enhances melanoma proliferation	Stecca et al., 2007
			Methylated in pancreatic cancer	Martin et al., 2005
			Decreases VEGF-mediated tumor angiogenesis	Agrawal et al., 2017
			Down-regulated during tumor angiogenesis in human tumors	Olsen et al., 2004
IGF1	1.458	Axon guidance, fibrosis	Enhances survival of breast cancer brain metastasis.	Palmieri, 2012
			IGF promotes EMT and stemness via NANOG and STAT3 signaling (melanoma brain metastasis)	DeVita et al., 2012; Yao C. et al., 2016
			IGF-1 supports tumor proliferation in melanoma metastasis.	Capoluongo, 2011
GAB1	1.489	Axon guidance, neuropathic pain, phagosome formation, Tec kinase, leukocytes, role of macrophages, fibroblasts, and endothelial cells, Rho family, thrombin signaling, aldosterone signaling	Mediates tumor angiogenesis in melanoma through Akt and ERK1/2 pathways.	Zhao et al., 2011
			GAB1 upregulation promotes tumor cell invasion, migration via CXCR4 and tumor growth.	Vasyutina et al., 2005; Seiden-Long et al., 2008; Sang et al., 2015
			GAB1 enhances vascular sprouting via VEGF-induced eNOS activation.	Lu et al., 2011
GNA14	1.935	Axon guidance, ephrin receptor signaling, Tec kinase, Rho family, thrombin signaling	Inhibits cell differentiation and enhances tumorigenicity after TNFa stimulation	Oshima et al., 2014
			Activates STAT3 signaling, NF-kB and Ras-dependent pathways	Liu and Wong, 2005; Kwan et al., 2012; Lee et al., 2012
CXCR4	2.175	Axon guidance, ephrin receptor signaling, leukocytes	Highly expressed in brain metastases across cancer types (breast, lung, kidney, colon, ovary, prostate, and thyroid).	Salmaggi et al., 2009
			Activation of CXCR4-CXCL12 axis enhance tumor cell migration.	Chung et al., 2017
			CXCR4 disrupts vascular permeability through PI3K-AKT and FAK pathway.	Weidle et al., 2016
			CXCR4 induces growth and metastasis of cancers including melanoma regardless of organ specificity	Kim et al., 2006; Burnett et al., 2015
RAC2	2.711	Axon guidance, ephrin receptor signaling, leukocytes	Rac2 promotes melanoma metastasis and tumor angiogenesis.	De et al., 2009; Joshi et al., 2014
			Rac2 activates SDF-1/CXCR4 system	Shen et al., 2012
GRIA4	-1.966	Neuropathic pain	GLUR4 knockdown enhances cell viability and proliferation, while stimulating cancer cell migration (through upregulation of MMP2 and integrins)	Luksch et al., 2011
			Found to be hypermethylated in cancers (follicular lymphoma/oropharyngeal squamous cell carcinoma) compared to benign lesions	Kostareli et al., 2013
			GluR4 signaling activates MAPK and K-ras signaling (decreasing threshold of K-ras induced oncogenic signaling) in pancreatic cancer lesions	Herner et al., 2011
			Downregulated in glioma cells.	Savaskan et al., 2011
RND2	-1.746	Phagosome formation, Rho family, thrombin signaling	Downregulated in melanoma compared to melanocytes	Wen et al., 2017
			Enhances melanoma susceptibility to low dose cisplatin treatment through PARP cleavage and activation.	Ho et al., 2012
			Aza treatment sensitizes melanoma cells to therapeutics by upregulating RND2.	Halaban et al., 2009
RHOV	2.88	Phagosome formation, Tec kinase, Rho family, thrombin signaling	Overexpressed in lung cancer and NSCLC lines	Wang et al., 2017
			Signal transducer for PAK6, protein kinase implicated in prostate cancer chemoresistance	Shepelev and Korobko, 2012
JAK3	1.468	Tec kinase	Activates STAT5A	Sharfman, 2012
			Activates PD-L1 antitumor suppression in melanoma cells	Jiang et al., 2013
			Promotes squamous cell carcinoma migration and proliferation	Zhang et al., 2017
			Expressed at higher levels in brain metastases of melanoma compared to primary melanoma	Kesari et al., 2015
FRK	1.627	Tec kinase	Causes PTEN deregulation and is overexpressed in melanoma	Bosserhoff, 2017
			Enhances tumor progression in cell-type specific manner	Zhao et al., 2011
STAT5A	2.211	Tec kinase	Enhances cell viability, proliferation, and growth.	Mirmohammadsadegh et al., 2006; Hassel et al., 2008; Liao et al., 2010
			STAT5a inhibition enhances cisplatin susceptibility.	Zhang, 2012
			Activated in melanoma cells by EGFR and JAK1 and SRC	Rochman et al., 2010
			Active STAT5a signaling induces EMT and CSC markers and promotes metastasis in prostate cancer	Schmidt et al., 2014
PIP5KL1	-1.805	Aldosterone signaling, Rho family	PIP5KL1 upregulation suppresses tumor cell proliferation, migration, and growth.	Shi et al., 2010a,b
			Overexpression of PIP5KL1 markedly inhibited (*P* < 0.05) serum-induced phosphorylation of AKT1	Shi et al., 2010b
			PIP5KL1 transduction induces apoptotic changes in 293T cells	Wang et al., 2006
GATA3	-1.525	Thrombin signaling	GATA3 suppression promotes EMT, metastasis and suppresses cell differentiation and alters the tumor microenvironment by inducing angiogenesis in breast cancer.	Chou et al., 2010, 2013
			Genomic analyses reveal a high frequency target mutation of GATA3 in breast cancer, melanomas, clear cell sarcoma and poor prognosis.	Miettinen et al., 2014; Takaku et al., 2015
			GATA3 is a negative regulator of tumor invasion and growth.	Takaku et al., 2015
			Ectopic GATA3 ablates canonical TGF-β-Smad signaling axis.	Sun et al., 2013
DKK1	-4.852	Role of macrophages, fibroblasts, and endothelial cells	DKK1 is hypermethylated in several cancer types.	Mazon et al., 2016
			^3^ DKK1 protein secretion abrogated in melanoma however reported in breast, prostate and lung cancer lines.	Kuphal et al., 2006; Forget et al., 2007
			^4^ DKK1 suppresses melanoma cell invasion.	Chen et al., 2009
			^5^ Temsirolimus (mTOR inhibitor) potentiates temozolomide (second line treatment for brain cancers) in metastatic melanoma by DKK1 pathway.	Niessner et al., 2017
FRZB	-1.783	Role of macrophages, fibroblasts, and endothelial cells	Hypermethylated in melanoma and methylation promotes melanoma migration and invasion.	Ekström et al., 2011; Micevic et al., 2017
			FRZB reverses EMT in pancreatic cancer.	Zi et al., 2005
IL32	-1.746	Role of macrophages, fibroblasts, and endothelial cells	IL-32α administration prevents human melanoma proliferation through p21, p53 and TRAILR1.	Nicholl et al., 2016
			STAT1 and IL-32 signaling mediates immunoresponse upon TLR2/6 agonists and IFN-gamma treatment in melanoma.	Mauldin et al., 2014
IRAK2	1.696	Role of macrophages, fibroblasts, and endothelial cells	IRAK2 and LAMP1 are negatively regulated by miR-373.	Farooqi et al., 2015
			Novel role of IRAK2, RAF6, and Ras in p38 MAPK activation.	McDermott and O’Neill, 2002
			Highly expressed in malignant melanomas.	Li et al., 2009
C5	1.528	Role of macrophages, fibroblasts, and endothelial cells	C5a promotes the development and growth of melanoma (targeted melanoma therapy using complement-inhibitory drugs).	Nabizadeh et al., 2013
			C5a inhibition blocks tumor progression and angiogenesis.	Corrales et al., 2012; Nitta et al., 2013
CLDN1	-2.537	Leukocytes	Claudin-1 acts as a metastasis suppressor, and loss of claudin-1 is positively correlated with poor outcomes in lung adenocarcinoma.	Chao et al., 2009
			Claudin-1 prevents brain metastasis of melanoma.	Izraely et al., 2015
			Decreased expression in vessels associated with melanocytic neoplasms.	Cohn et al., 2005; Leotlela et al., 2007
CTNNA2	-1.483	Leukocytes	Deleted in MEL10 and predicted to be a tumor suppressor.	Fanjul-Fernández et al., 2013; Ding et al., 2014


We utilized Gene Set Enrichment Analysis (GSEA) to refine this dataset and identify genes with common biological functions based on curated datasets (Subramanian et al., 2005). Using directional *p*-values for differential expression in the Stroke-MET transcriptome, we identified 50 hallmark gene sets, of which 21 were upregulated and 29 were downregulated. At an FDR < 0.05, 7 upregulated and 15 downregulated gene sets were significantly enriched in Stroke-MET (Figure 6D,E). The gene-set enrichment analysis indicated an enriched up-regulation of PI3K-mTOR pathway (Sarris et al., 2012) and downregulation of p53 pathway (Oren, 2003) often associated with tumor progression in cancer. These analyses of gene classes and identification of specific genes uniquely expressed in stroke-melanoma metastasis vs. “general” brain metastasis identifies many that are associated with neuronal pathfinding, neuronal tropic and trophic interactions and neuronal cell motility, supporting a role of the metastatic cell in an analogous position in the regenerative neurovascular niche to the normal principle cell in this niche, the neuroblast.

### Mimicking Stroke-Like Conditions With Oxygen-Glucose Deprivation (OGD) Facilitates Brain Metastasis

To model the influence of stroke on melanoma metastasis *in vitro*, we assessed the (a) metastatic properties and (b) molecular profile of melanoma cells in stroke-like conditions using a well-established *in vitro* model: OGD (Hillion et al., 2005). Metastatic cells were treated with conditioned media harvested from human brain endothelial cells (h-EC) or human astrocytes (h-Astro) subjected to either OGD or control conditions of normal oxygen and glucose levels.

### Oxygen Glucose Deprivation (OGD) Enhances Chemoattraction of Melanoma Cells by Brain Cells

#### Oxygen Glucose Deprivation (OGD) Enhances Migration of Melanoma Cells

**FIGURE 7:**
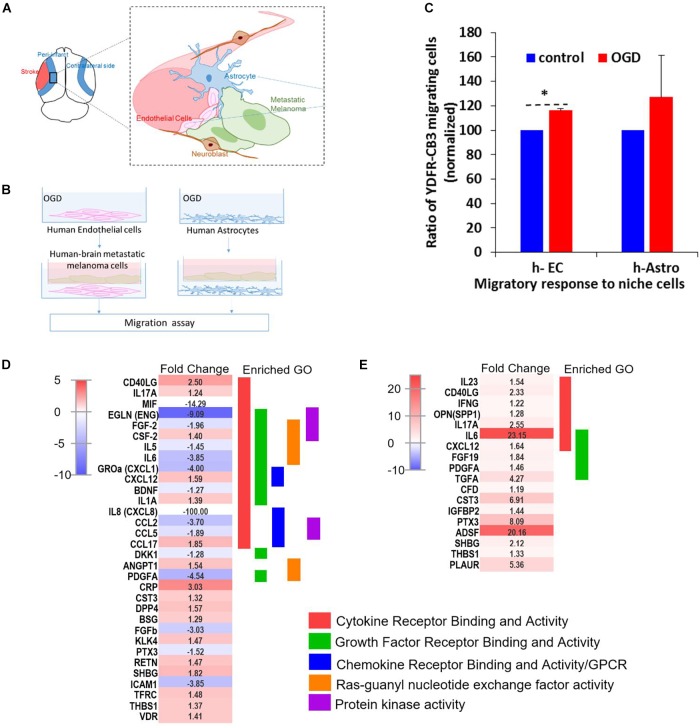
OGD alters chemokine/cytokine responses in the neurovascular niche and enhances migration of brain metastatic cells. **(A)** Schematics showing the interaction of human astrocytes (h-Astro) and human endothelial cells (h-EC) with metastatic melanoma cells. **(B)** Schematics of method of oxygen glucose deprivation (OGD) conditioned media exposed to metastatic melanoma cells. **(C)** Migratory response of metastatic melanoma cells exposed to OGD conditioned media from cells are shown as mean ± SD, ^∗^*P* < 0.05. **(D,E)** Heatmap of relative expression of secreted proteins profiled from the medium of h-Astro **(E)** and h-EC **(D)**. Functionally grouped enriched pathways are shown in one color. Enrichment/depletion tests performed with ClueGO application on cytoscape, κ score ≥ 0.4.

**FIGURE 8:**
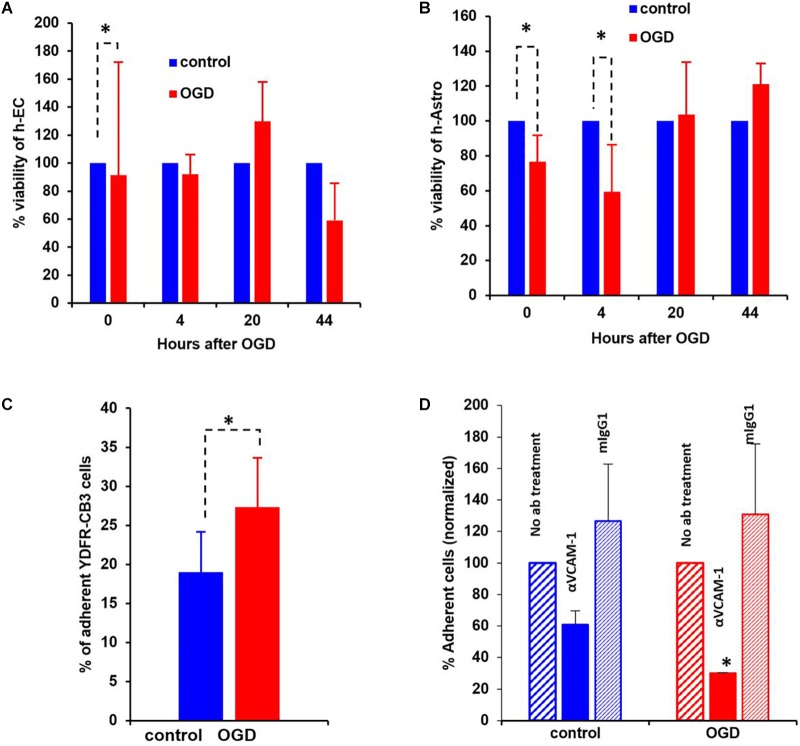
Oxygen-glucose deprivation (OGD) affects viability of human-endothelial cells (h-EC), astrocytes (h-Astro) and adhesion of metastatic melanoma cells. **(A,B)** Percentage viability of h-EC **(A)** and h-Astro **(B)** subjected to OGD for 4 h followed by a 4, 20, and 44 h reperfusion phase. Cell viability as measured by XTT assay. The bars represent the average % viability of OGD exposed cells, normalized to control cells subjected to normal oxygen and glucose levels, mean ± SD, ^∗^*p* < 0.05. The different time points indicate the hours in reperfusion after OGD/control conditions. **(C)** h-EC were subjected to OGD for 4 h followed by a 20 h reperfusion phase. m-Cherry expressing macro- and micro-metastatic melanoma cells were seeded on top of the h-EC monolayer and incubated for 30 min, to allow adhesion to occur. The fluorescence signal of labeled cells was measured before and after removal of non-adherent cells. The bars represent the % adherent cells in the wells. The graph represents an average of three independent experiments + SD. ^∗^*P* < 0.05. **(D)** m-Cherry expressing metastatic melanoma cells were seeded on top of the OGD or control h-EC monolayers and incubated for 30 min with 5 μg/mL VCAM-1 blocking Ab, an isotype control (mIgG1), or without IgG. The fluorescence signal of labeled cells was measured before and after removal of non-adherent cells. The bars represent % adherent melanoma cells seeded with anti-VCAM-1 blocking Ab or isotype control normalized to % adherent melanoma cells seeded without Ab. The graph represents an average of two independent experiments + SD. ^∗^*p* < 0.05.

Since cell migration is an essential component of the metastatic cascade (van Zijl et al., 2011), we examined the directed migratory capacity of brain metastatic melanoma in response to cues from h-EC and h-Astro after OGD and control conditions. Melanoma cells were added upon a collagen-coated Transwell insert and allowed to migrate toward h-EC/h-Astro subjected to OGD or control conditions. While melanoma cells displayed significantly increased migration toward OGD-exposed h-EC compared to control conditions (*p* < 0.05), OGD-conditioned media from h-Astro did not significantly alter migration (Figure 7C). OGD exposed h-EC also displayed enhanced adhesion of metastatic melanoma cells, which was reduced by blocking VCAM-1 (Figure 8). Thus, metastatic characteristics in melanoma cells can be enhanced by recapitulating stroke-like conditions *in vitro*, and this *in vitro* results suggest that endothelial cells are key mediators of regeneration-potentiated metastasis.

#### OGD Alters Cytokine/Chemokine Responses in Astrocytes and Endothelial Cells *in vitro*

We next examined the secreted factors from OGD-exposed endothelial cells and astrocytes to determine signaling cues that play a role in the *in vitro* migration findings, and isolate the influence of individual cell types on metastatic cells. To identify these candidate factors, secreted and extracellular molecules from h-EC (OGD vs. control) and h-Astro (OGD vs. control) were measured using a cytokine array. Thirty-two cytokines (fold change < -1.2 or > 1.2) were differentially secreted from OGD vs. control h-EC and 19 were differentially secreted from OGD vs. control h-Astro (Figure 7D,E). To identify gene ontologies in these *in vitro* stroke-endothelial and stroke-astrocyte secretomes, enrichment depletion (simultaneous analysis of enriched: over-represented DE genes and depleted: under-represented genes between two states) (Rivals et al., 2007) was performed for both cellular processes and molecular functions. Both endothelial and astrocyte-derived OGD secretomes were enriched for cytokine and growth factor receptor activity while endothelial-derived secretomes displayed additional enrichment of chemokines, Ras-GTP, and protein kinase activity, further supporting the central role of endothelial cell in contributing to this pro-metastatic niche.

#### OGD Reduces Viability of Human Brain Endothelial Cells and Astrocytes

The viability of neurovascular niche cells was assessed under OGD conditions. Human brain h-EC and h-Astro were subjected to OGD or control (normoxic) conditions for 4 h followed by 4, 20, and 44 h of normoxia recapitulating reperfusion. Cell viability was then measured by XTT assay. The results showed that h-Astro were more sensitive to OGD than h-EC (Figure 8A,B). While the viability of the h-Astro was significantly reduced after 4 h of OGD conditions (*t* = 0, *p* < 0.05), a significant reduction in h-EC viability only occurred after 44 h (*p* < 0.05). When the normoxic phase was prolonged, “stroked” astrocytes showed a gradual recovery over time (20 and 44 h after start of the reperfusion phase). These findings mimic spontaneous recovery of neurovascular components observed after stroke.

#### OGD Enhances the Adhesive Capacity of h-EC to Metastatic Melanoma Cells

A crucial initial step in melanoma cell transmigration through the brain vasculature is the adhesion of cancer cells to the endothelium. To investigate whether OGD enhances the ability of h-EC to bind melanoma cells, we compared the adhesion of brain-metastasizing melanoma cells to OGD and control h-EC. The brain-metastasizing melanoma cells showed significantly greater adherence to OGD exposed h-EC than to control (Figure 8C,D). This result suggests that stroke-like conditions recapitulated by OGD facilitates the formation of brain metastasis by making h-EC more amenable to tumor cell adhesion. The expression of the endothelial cell adhesion molecules ICAM-1 and VCAM-1 was tested in control and OGD exposed h-EC. Flow cytometry analysis demonstrated a slight up-regulation in the expression of both molecules following OGD (data not shown). To further demonstrate the increased adherence of h-EC to metastatic melanoma cells, YDFR-CB3m-Cherry cells were co-cultured with h-EC and the above experiment was similarly performed in the presence of a VCAM-1 blocking antibody. Inhibiting VCAM-1 significantly decreased OGD (and not control) h-EC adherence to YDFR-CB3m-Cherry suggesting its importance in brain metastasis in the regenerative neurovascular niche (Figure 8D).

### Transcription Landscapes From Regenerative Niche and Melanoma Brain Metastasis: *in vivo* and *in vitro* Overlaps

To delineate the influence of specific niche cells (endothelial/astrocytes) upon the metastatic transcriptome *in vitro*, we sequenced the total RNA from metastatic melanoma in conditioned media harvested from h-EC or h-Astro subjected to OGD or controls. The differentially expressed (DE) genes from OGD_EC_-MET/OGD_Astro_-MET were compared with the transcriptional *in vivo* profile of regeneration-potentiated melanoma (Stroke-MET) to determine overlap between the *in vivo* and *in vitro* studies.

**FIGURE 9:**
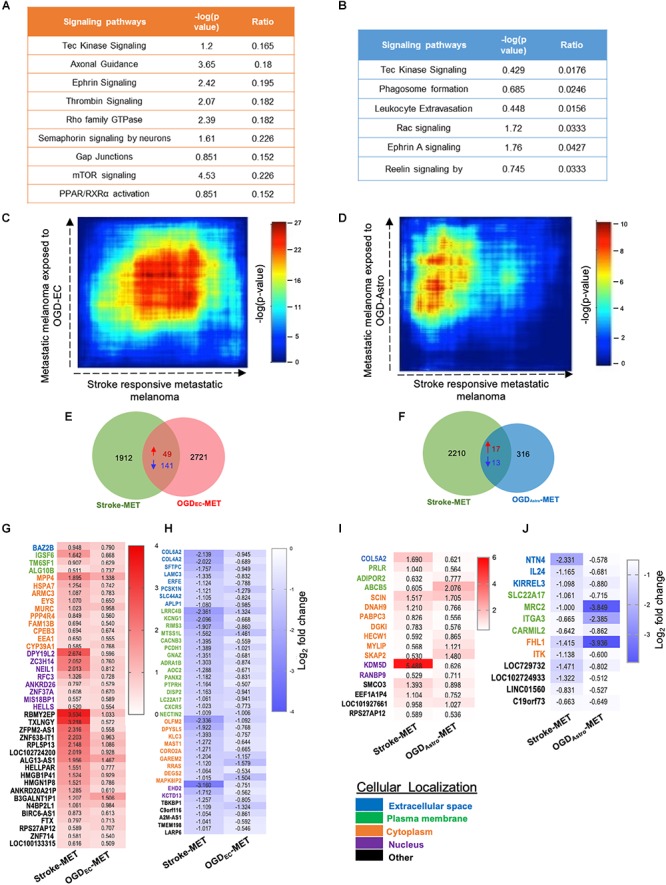
Overlapping transcription landscapes from regenerative NV niche after stroke and melanoma brain metastasis. **(A,B)** Common canonical signaling pathways between the *in vivo* Stroke-MET and *in vitro* OGD-_EC_MET/OGD-_Astro_MET transcriptome. **(C,D)** Rank–rank hypergeometric overlap (RRHO) analysis of common genes expression spread between Stroke-MET and OGD-_EC_MET/OGD-_Astro_MET transcriptome. RRHO analysis of common genes expression spread between Stroke-MET and OGD-_Astro_MET datasets. Red and blue pixels on the heatmap depict a high and low number of overlapping genes, respectively. Stroke-MET and OGD_EC_-MET display stronger hypergeometric overlap those with OGD_Astro_-MET transcriptome. **(E,F)** Overlapping up and downregulated genes between *in vivo* Stroke-MET and *in vitro* OGD-_EC_MET/OGD-_Astro_MET transcriptome are depicted in the Venn diagram. **(G–J)** Heatmap of log_2_ FC of overlapping genes between Stroke-MET and OGD-_EC_MET/OGD-_Astro_MET transcriptome. The up and down regulated overlapping genes are shown in red and blue a change in gradients of these color correspond to the level of log_2_ FC as shown on the scale bars. Genes classified based on the cellular location and the color coded as shown in the key at the bottom of the heatmaps : Blue: extracellular, green: plasma membrane, orange: cytoplasm, violet: nucleus, and black: other.

We performed a rank–rank hypergeometric overlap (RRHO) that compares the ranked order and the log_10_-transformed *t*-test *p*-value of *in vitro* differentially expressed genes to *in vivo* differentially expressed genes in this melanoma cell line, and in stroke conditions. The heat maps show that top ranked genes in OGD_EC_-MET overlapped with those of Stroke-MET to a greater extent than OGD_Astro_-MET (Figure 9C,D). An assessment of overlapping canonical signaling systems reveals that the top highly up/down-regulated pathways all play important potential or predicted roles in governing melanoma metastasis: PPAR/RXRα, angiopoietin signaling, mTOR, Semaphorin signaling between the Stroke-MET and OGD_EC_-MET transcriptome, and ephrin receptor signaling in both Stroke-MET and OGD_Astro_-MET transcriptomes (Figure 9A,B). This analysis indicates that the *in vitro* melanoma profile, when exposed to stroke-conditioned endothelial cells, significantly overlaps with the *in vivo* melanoma gene expression profile in the stroke regenerative neurovascular niche, but not that of melanoma exposed to stroke-conditioned astrocytes.

To move forward on this similarity, a comparison of genes similarly up and down regulated genes in *in vivo* regeneration-potentiated melanoma transcriptome (Stroke-MET) and *in vitro* OGD_EC_-MET transcriptome showed 343 overlapping significant genes (*p* < 0.05) of which 49 were upregulated and 141 were downregulated. Stroke-MET transcriptome compared to OGD_Astro_-MET transcriptome showed 45 significant genes, of which 17 were upregulated and 13 were downregulated (Figure 9E–J). This gene list is small and provides a tractable set of genes for mechanistic study in their effect on tumor metastasis and stroke neurovascular responses.

**FIGURE 10:**
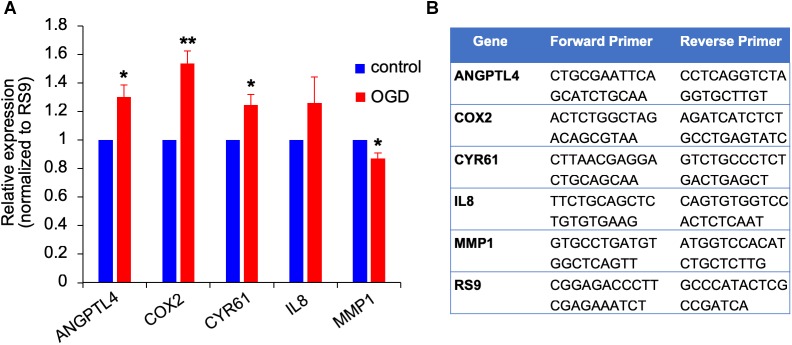
Gene expression changes in OGD exposed metastatic melanoma cells. **(A)** The effect of OGD exposed astrocyte secreted factors on melanoma gene expression. Astrocytes were subjected to OGD or control conditions for 4 h followed by a 20 h reperfusion phase. Metastatic melanoma cells were treated for 24 h with the OGD or control conditioned media collected from the reperfusion phase. Total RNA was extracted and gene expression was determined by RT-qPCR. mRNA expression levels were normalized to the expression of RS9. Bars represent gene expression in cells treated with conditioned medium of “stroked” astrocytes normalized to gene expression in cells treated with control astrocyte conditioned medium mean ± SD of three independent experiments, ^∗^*p* < 0.05, ^∗∗^*p* < 0.01. **(B)** Primer sequences used for the qPCR analysis shown in **(A,B)**.

We previously showed that brain metastasizing melanoma express a unique set of genes distinct from that found in cutaneous melanoma (Izraely et al., 2012). Some of these genes are known as metastasis-mediating genes. Our objective was to examine whether the expression of this set of genes is altered in melanoma cells subjected to *in vitro* post-stroke reperfusion conditions, i.e., to supernatants of “stroked” astrocytes. We incubated “stroked” and control astrocytes in starvation medium for 20 h to allow secretion of post-stroke soluble factors into the medium, collected these supernatants, and added them into brain metastatic melanoma cultures. We observed an upregulation in the expression of ANGPTL4, COX2, and CYR61, and a slight downregulation in MMP1 expression. These results suggest that stroked astrocytes secrete factors that may induce the metastatic potential of melanoma cells (Figure 10).

**FIGURE 11:**
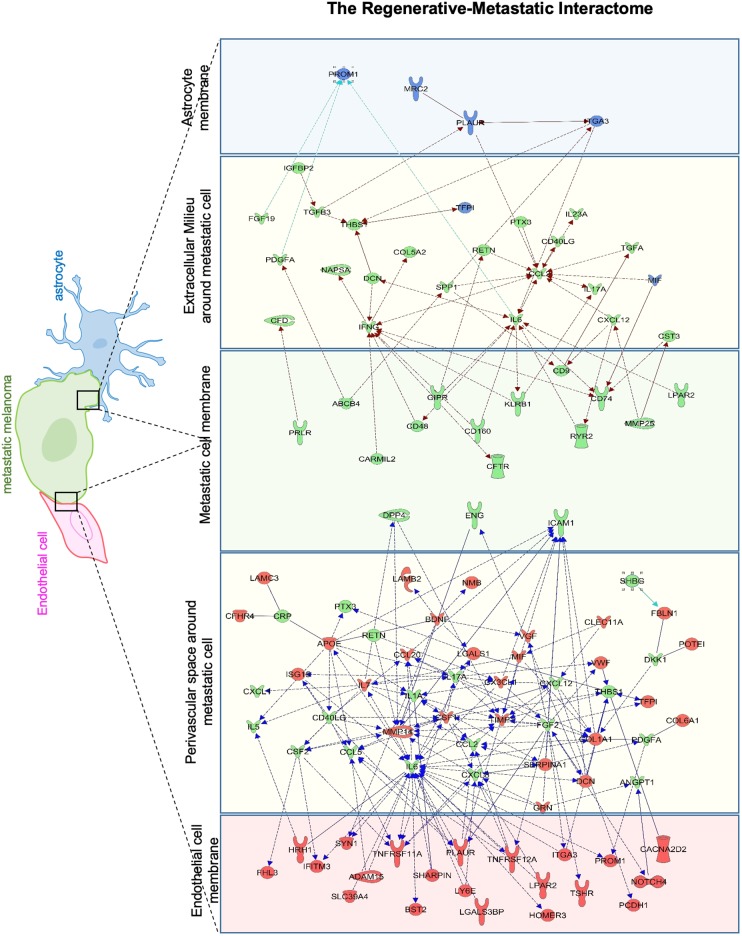
Astrocyte–metastatic cell and endothelial cell–metastatic cell interactome. Extracellular and plasma membrane bound proteins were chosen to determine putative gene interactions between metastatic melanoma cell with two cell types in the regenerative neurovascular niche–astrocyte/endothelial cells. Astrocyte–metastatic cell interactome display differentially expressed proteins in blue shows OGD exposed astrocyte secretome (blue panel) and their interactions with Genes in the metastatic melanoma cells from the Stroke-MET transcriptome colored in green (green panel). Endothelial cell–metastatic cell interactome differentially expressed proteins in red shows OGD exposed astrocyte secretome (red panel) and their interactions with genes in the metastatic melanoma cells from the Stroke-MET transcriptome colored in green (green panel). Yellow panels depicts molecules in the extracellular space. Molecules in yellow: secretome, red: OGD exposed endothelial cells, blue: OGD exposed astrocytes, green: stroke responsive melanoma Stroke-MET.

To identify putative molecular interactions that facilitate metastasis in stroke-like conditions, we profiled membrane and secreted proteins between endothelial cells/astrocytes, and metastatic melanoma in the regenerative neurovascular niche. An interaction map was generated of putative interactions between regeneration-potentiated melanoma cells and the two cells in the regenerative neurovascular niche that were determined to have a demonstrated *in vitro* effect on melanoma metastasis–astrocytes and endothelial cells. This approach used significantly regulated genes in *in vivo* regeneration potentiated melanoma that were membrane-bound or secreted, and had signaling partners on another cell type: astrocyte–melanoma and endothelial cell-melanoma exposed to OGD *in vitro*. A greater number of molecular interactions between endothelial cells and metastatic melanoma were identified compared to astrocytes. Interestingly PROM1 a surface marker of tumor initiating cancer cells (Raso et al., 2011) was found in both interactomes. IL-6 and CCL2 had the most number of signaling partners in both the interactomes. These putative molecular interactions can be used to explore the functional significance of target molecules, and characterize the dynamic interactions that occur between metastatic cells and the regenerative neurovascular niche to drive regeneration-potentiated metastasis (Figure 11).

### Network Analysis of Prognostic Gene Expression Profiles Identify Key Central Mediators

**FIGURE 12:**
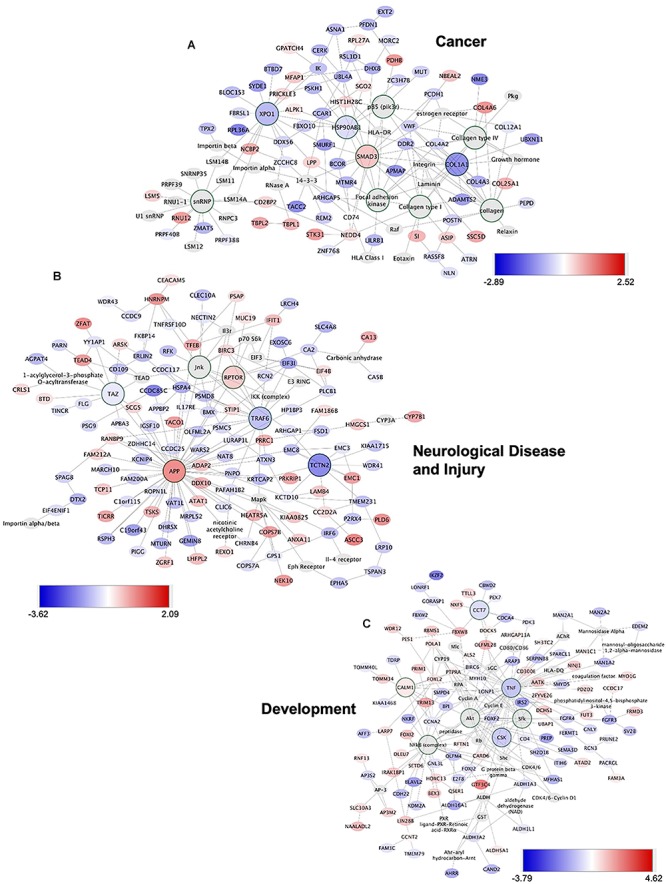
Gene network analysis of highly repetitive classes of coordinated diseases and functions from the Stroke-MET transcriptome. Network of gene interactions representing genes associated with “cancer” **(A)**, “Neurological disease and injury” **(B)**, “Development” **(C)**. Red: upregulated genes, blue: downregulated genes, and larger nodes: central and highly connected genes. Nodes shown in gradients of red and blue are up- and down-regulated, respectively. Centrality values are provided in the Table 2. Central genes with higher connectivity with neighboring genes are shown as larger nodes in each category.

To gain insights into system-level properties of biologically important genes, the significantly regulated genes in regeneration-potentiated melanoma (Stroke-MET) were grouped into networks and assigned focus scores based on *p*-values. Using network analysis allows us to identify genes of a high functional and biological significance in the metastatic cells within the regenerative niche. Focus scores indicate the likelihood of genes within a network being found together, taking into account the size of the network based on relevance to disease or function (Long et al., 2004). The genes with specific connectivity belonging to disease classes from the Stroke-MET transcriptome with highest focus scores were used for centrality analysis (Cytoscape, CytoNCA). Centrality analysis identifies genes that are highly interconnected and influence a large number of other genes within a network. We identified these central genes from three classes of gene networks with coordinated functions and diseases including neurological disease and injury, development, and cancer (Tang et al., 2015). We determined the degree and betweenness centrality of the central genes, which are designated by large node size in the networks (Figure 12A–C). A gene’s degree and betweenness centrality represents its interconnectedness to other genes in the network, and its influence over adjacent genes, respectively (Ozgur et al., 2008). Centrality values are provided in Table 2. From these networks we identified APP, RPTOR, and TAZ (neurological injury network), extracellular matrix signatures such as collagen I, IV and FAK (cancer network) as central genes that are predicted master regulators specific to brain metastasis and potential targets for further experimental analysis.

**Table 2:** Centrality values of differentially regulated genes.

Disease Network Centralities			
	Degree	Eigenvector	Betweenness
**Cancer**			
XP01	17	0.08596991	3300.861
SMAD3	16	0.3436861	3565.8477
snRNP	15	0.012245474	2225.245
collagen	15	0.32417372	1237.3102
Collagen type I	12	0.31365713	1503.7742
Focal adhesion kinase	11	0.26898885	1088.7654
COL1A1	11	0.3225594	504.3585
Collagen type IV	10	0.24174887	657.4651
HSP90AB1	9	0.12958206	1337.7277
p85 (pik3r)	9	0.16082734	1101.0994
**Neur. Disease and Injury**			
APP	56	0.6285145	14092.723
TRAF6	17	0.26952243	2496.349
Jnk	14	0.19043593	2337.5176
TAZ	11	0.11805606	2943.962
TCTN2	10	0.03490787	1265.8921
RPTOR	9	0.18103331	1985.5358
**Development**			
TNF	48	0.5610349	11893.423
NFkB (complex)	23	0.28850913	4632.912
Akt	21	0.30290568	5351.877
CALM1	11	0.12641135	1627.4298
Sfk	9	0.21849017	400.375
CSK	9	0.16786034	1086.8019
CCT7	9	0.02809526	1204.9857


**FIGURE 13:**
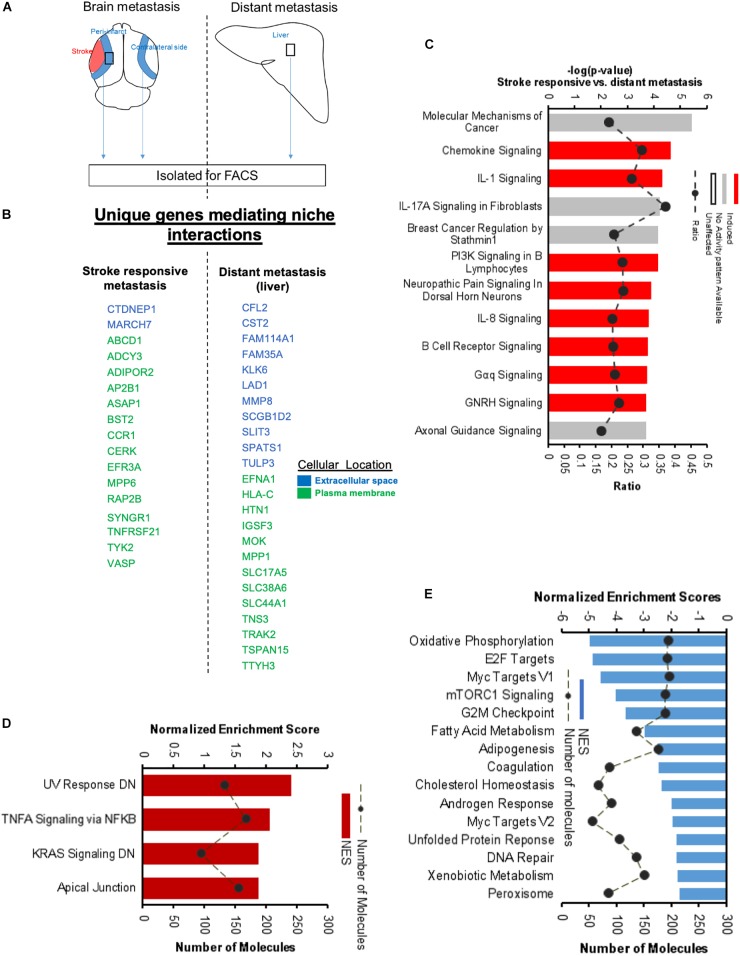
Unique molecular signaling systems in brain metastasis. **(A)** Schematic representation of brain and distant sites of metastasis compared. The significant and differentially expressed genes between Brain metastasis (stroke responsive metastatic cells) and distant metastasis (liver metastasis) were compared. **(B)** Unique extracellular and plasma membrane genes that interact with the surrounding niche in each of these two transcriptomes were identified at FPKM values > 4. **(C)** Top canonical pathways differentially regulated in brain metastatic melanoma over a distant metastatic site (liver) with the largest negative logP values are shown. The dashed line represents the ratio of the number of molecules in the dataset over the number of molecules in the pathway. **(D,E)** GSEA of hallmark genes up and downregulated in the differentially expressed genes of brain metastatic vs. liver metastasis.

To further identify the unique molecular profile of metastatic melanoma cells within the regenerative neurovascular niche after stroke, we compared the differentially expressed genes of these cells in this niche to those in metastatic melanoma cells outside of this niche, in liver metastasis. To identify biological variation without interference from different isoform abundance, we determined differences at the gene-level that are identifiable based on a RefSeq model (Soneson et al., 2015). FPKM values above 4 were used as a cut off to identify unique molecules in brain metastasis and liver metastasis (Figure 13A,B). While gene-level overlap tests for overall changes in transcriptional output of a gene, individual transcripts may have different mechanisms or effects on compared conditions. To further to detect these changes in transcript isoforms, we used the Ensembl model at the transcript-level (Conesa et al., 2016). A core comparison analysis showed that top canonical pathways are composed of chemokines and cytokines in the brain compared to the liver metastasis (Figure 13C). Interestingly, genes and pathways involved in axon guidance and neuropathic pain are significantly enriched in the brain metastasis compared to the liver. GSEA analysis of hallmark gene sets show 4 upregulated and 15 downregulated pathways significantly enriched in the differentially expressed transcriptome of Stroke-MET vs. liver-metastasis (Figure 13D,E). Brain metastasis to the regenerative neurovascular niche exhibits a unique signaling signature involving repair pathways not observed in distant metastasis.

## Discussion

The dynamic cross-talk between cancer cells and neural niche elements provides insight into the influence of the tumor microenvironment on metastasis. Neuronal precursor cells, astrocytes, and endothelial cells have all been found to play a crucial role in creating a permissive niche that drives metastatic success (Psaila and Lyden, 2009; Venkatesh et al., 2015). In both neural injury and brain metastasis, the local microenvironment undergoes analogous cellular and molecular changes that influence cell proliferation, differentiation, and survival (Chen B. et al., 2009; Termini et al., 2014). Both tumor progression and regenerative tissue reorganization require angiogenic vasculature, co-localization of a progenitor or tumor cell, and dynamic cell–cell signaling (Ohab et al., 2006; Prakash and Carmichael, 2015; Carmichael, 2016). In this study, we provide compelling evidence that cues emanating from the regenerative neurovascular niche facilitate brain melanoma metastasis.

The present data indicate that cellular and molecular features of the regenerative neurovascular niche induce the preferential localization of melanoma to the peri-infarct over other brain regions independent of changes in cerebral blood flow, blood–brain barrier breakdown, and vascular permeability normally found after stroke. Metastatic melanoma localized specifically to regions on angiogenic blood vessels closely associated with neuroblasts. However, angiogenic vessels alone were not sufficient to produce the significant amount of brain metastasis seen in stroke. This differential localization of metastatic melanoma to blood vessels associated with migratory neuroblasts identifies a central cellular complex in the regenerative neurovascular niche that enhances melanoma metastasis. After the initial localization of metastatic cells to neuroblast-associated angiogenic vessels, there is a progressive increase in spatiotemporal associations between neuroblasts and melanoma. Interestingly, we confirmed that neuroblasts interact with metastatic melanoma cells in human brain melanoma metastases. Taken together, these findings point to a novel, potentially crucial role of neuroblasts in brain metastasis raising new questions as to how neuroblast–melanoma interactions facilitate brain metastasis on a cellular and molecular level.

Brain endothelial cells and astrocytes are key supportive elements involved in repair processes within the regenerative neurovascular niche after stroke that are vital for efficient metastasis (Ohab et al., 2006; Langley and Fidler, 2013). The regenerative neurovascular niche significantly enhances melanoma-vasculature interactions *in vivo*, providing a compelling role for endothelial cells in mediating this regeneration-potentiated metastasis. Metastatic melanoma cells within this specialized niche also modify phenotypic structure of the surrounding vasculature. Interactions with astrocytes occur after extravasation but the course of astrocytic interactions is unaltered in the presence of the regenerative neurovascular niche. Thus cross-talk between melanoma and the specialized vasculature that is associated with neuroblasts governs brain metastasis in the regenerative neurovascular niche.

To further tease out the influence of stroke repair on the metastatic transcriptome, we isolated regeneration-potentiated melanoma cells and compared these to brain metastatic melanoma cells outside of the regenerative neurovascular niche using RNAseq. We identify 22 differentially regulated genes in regeneration-potentiated metastatic cells that activate molecular programs strongly implicated within metastasis, including established oncogenes such as JAK3, STAT5A, RHOV, and RAC2.

To isolate molecular interactions between metastatic cells and individual cells in the regenerative neurovascular niche, we mimicked stroke-like conditions *in vitro* using OGD. Metastatic migration was significantly enhanced in response to OGD-exposed endothelial cells while the contribution from astrocytes was not significant, supporting our *in vivo* observations that endothelial cell interactions with melanoma are key to governing metastasis. We compared the transcriptomes of *in vivo* regeneration-potentiated melanoma cells with *in vitro* melanoma cells exposed to supernatant from OGD-exposed astrocytes and endothelial cells. *In vivo* regeneration-potentiated metastatic cells showed greater overlap in transcriptional profile with metastatic melanoma exposed to OGD-endothelial cell supernatants than cells exposed to OGD-astrocyte supernatant, suggesting that endothelial cells are the pivotal cell type in guiding regenerative-metastatic signaling. This parallels the central role of vasculature noted above in governing metastatic cell localization to the regenerative neurovascular niche.

We identified molecular interactomes between secreted and extracellular proteins from the endothelial cells and astrocytes to those on *in vivo* regeneration-potentiated melanoma cells. These include both established (IL23, IL17A) and novel (DPP4, CST3) candidates for signaling in metastasis. Molecular pathway analysis reveals central genes expressed by melanoma cells that might serve as hubs in organizing the pro-metastatic molecular response in regeneration-potentiated melanoma, such as APP, SMAD3, and RPTOR, supporting the hypothesis that stroke induces pro-metastatic alterations to the melanoma transcriptome.

## Conclusion

These studies demonstrate that (a) the regenerative neurovascular niche enhances metastasis by facilitating melanoma interactions with regenerative neurovascular niche components, (b) regeneration-potentiated melanoma cells display a unique transcriptome with an enhancement of genes conducive to metastasis, (c) mimicking repair such as stroke-like conditions successfully enhances metastasis while allowing for the identification of cell-specific signaling molecules that play important roles in driving metastasis. These molecular targets may allow promotion of recovery after stroke and brain disease without affecting cancer-related mechanisms.

## Author Contributions

RP, SI, DH, GC, IW, and SC conceptualized and designed the work. RP, SI, NT, RL, MR, OS-A, SB-M, TM, and SO collected the data. RP, SI, NT, RL, RK, DH, GC, IW, and SC analyzed and interpreted the data, and drafted and critically revised the manuscript. MR, OS-A, SB-M, TM, and SO analyzed the data. MM performed the experiments. RP, SI, NT, RL, RK, DH, GC, IW, SC, MR, OS-A, SB-M, TM, SO, and MM approved the final version of the manuscript to be published.

## Conflict of Interest Statement

The authors declare that the research was conducted in the absence of any commercial or financial relationships that could be construed as a potential conflict of interest.

## References

[B1] Abraham C. S. Harada N. Deli M. A. Niwa M. (2002). Transient forebrain ischemia increases the blood-brain barrier permeability for albumin in stroke-prone spontaneously hypertensive rats. *Cell. Mol. Neurobiol.* 22 455–462. 10.1023/A:1021067822435 12507394 PMC11533779

[B2] Agliano A. Martin-Padura I. Mancuso P. Marighetti P. Rabascio C. Pruneri G. (2008). Human acute leukemia cells injected in NOD/LtSz-scid/IL-2Rgamma null mice generate a faster and more efficient disease compared to other NOD/scid-related strains. *Int. J. Cancer* 123 2222–2227. 10.1002/ijc.23772 18688847

[B3] Agrawal V. Kim D. Y. Kwon Y. G. (2017). Hhip regulates tumor-stroma-mediated upregulation of tumor angiogenesis. *Exp. Mol. Med.* 49:e289. 10.1038/emm.2016.139 28127049 PMC5291840

[B4] Ben-Baruch A. (2008). Organ selectivity in metastasis: regulation by chemokines and their receptors. *Clin. Exp. Metastasis* 25 345–356. 10.1007/s10585-007-9097-3 17891505

[B5] Boiko A. D. Razorenova O. V. Swetter S. M. Johnson D. L. Ly D. P. Butler P. D. (2010). Human melanoma-initiating cells express neural crest nerve growth factor receptor CD271. *Nature* 466 133–137. 10.1038/nature09161 20596026 PMC2898751

[B6] Bosserhoff A. K. (2017). *Melanoma Development: Molecular Biology, Genetics and Clinical Application. 2017*. Cham: Springer International Publishing 10.1007/978-3-319-41319-8

[B7] Bronisz A. Godlewski J. Wallace J. A. Merchant A. S. Nowicki M. O. Mathsyaraja H. (2011). Reprogramming of the tumour microenvironment by stromal PTEN-regulated miR-320. *Nat. Cell Biol.* 14 159–167. 10.1038/ncb2396 22179046 PMC3271169

[B8] Brumm A. J. Carmichael S. T. (2012). Not just a rush of blood to the head. *Nat. Med.* 18 1609–1610. 10.1038/nm.2990 23135507 PMC4703036

[B9] Bullitt E. Aylward S. R. Van Dyke T. Lin W. (2007). Computer-assisted measurement of vessel shape from 3T magnetic resonance angiography of mouse brain. *Methods* 43 29–34. 10.1016/j.ymeth.2007.03.009 17720561 PMC2000457

[B10] Bullitt E. Gerig G. Pizer S. M. Lin W. Aylward S. R. (2003). Measuring tortuosity of the intracerebral vasculature from MRA images. *IEEE Trans. Med. Imaging* 22 1163–1171. 10.1109/TMI.2003.816964 12956271 PMC2430603

[B11] Bullitt E. Zeng D. Gerig G. Aylward S. Joshi S. Smith J. K. (2005). Vessel tortuosity and brain tumor malignancy: a blinded study. *Acad. Radiol.* 12 1232–1240. 10.1016/j.acra.2005.05.027 16179200 PMC2517122

[B12] Burnett R. M. Craven K. E. Krishnamurthy P. Goswami C. P. Badve S. Crooks P. (2015). Organ-specific adaptive signaling pathway activation in metastatic breast cancer cells. *Oncotarget* 6 12682–12696. 10.18632/oncotarget.3707 25926557 PMC4494966

[B13] Capoluongo E. (2011). Insulin-like growth factor system and sporadic malignant melanoma. *Am. J. Pathol.* 178 26–31. 10.1016/j.ajpath.11.004 21224039 PMC3069928

[B14] Carmichael S. T. (2016). Emergent properties of neural repair: elemental biology to therapeutic concepts. *Ann. Neurol.* 79 895–906. 10.1002/ana.24653 27043816 PMC4884133

[B15] Cavaglia M. Dombrowski S. M. Drazba J. Vasanji A. Bokesch P. M. Janigro D. (2001). Regional variation in brain capillary density and vascular response to ischemia. *Brain Res.* 910 81–93. 10.1016/S0006-8993(01)02637-3 11489257

[B16] Chao Y. C. Pan S. H. Yang S. C. Yu S. L. Che T. F. Lin C. W. (2009). Claudin-1 is a metastasis suppressor and correlates with clinical outcome in lung adenocarcinoma. *Am. J. Respir. Crit. Care Med.* 179 123–133. 10.1164/rccm.200803-456OC 18787218

[B17] Chen B. Friedman B. Cheng Q. Tsai P. Schim E. Kleinfeld D. (2009). Severe blood-brain barrier disruption and surrounding tissue injury. *Stroke* 40 e666–e674. 10.1161/STROKEAHA.109.551341 19893002 PMC2819286

[B18] Chen J. Li H. Chen H. Hu D. Xing Q. Ren G. Luo X. (2012). Dickkopf-1 inhibits the invasive activity of melanoma cells. *Clin. Exp. Dermatol.* 37 404–410. 10.1111/j.1365-2230.2011.04276.x 22420644

[B19] Chen Q. Khoury M. Chen J. (2009). Expression of human cytokines dramatically improves reconstitution of specific human-blood lineage cells in humanized mice. *Proc. Natl. Acad. Sci. U.S.A.* 106 21783–21788. 10.1073/pnas.0912274106 19966223 PMC2789167

[B20] Chou J. Lin J. H. Brenot A. Kim J. W. Provot S. Werb Z. (2013). GATA3 suppresses metastasis and modulates the tumour microenvironment by regulating microRNA-29b expression. *Nat. Cell Biol.* 15 201–213. 10.1038/ncb2672 23354167 PMC3660859

[B21] Chou J. Provot S. Werb Z. (2010). GATA3 in development and cancer differentiation: cells GATA have it!. *J. Cell Physiol.* 222 42–49. 10.1002/jcp.21943 19798694 PMC2915440

[B22] Chung B. Esmaeili A. A. Gopalakrishna-Pillai S. Murad J. P. Andersen E. S. Kumar Reddy N. (2017). Human brain metastatic stroma attracts breast cancer cells via chemokines CXCL16 and CXCL12. *NPJ Breast Cancer* 3:6. 10.1038/s41523-017-0008-8 28649646 PMC5460196

[B23] Chung K. Deisseroth K. (2013). CLARITY for mapping the nervous system. *Nat. Methods* 10 508–513. 10.1038/nmeth.2481 23722210

[B24] Cohn M. L. Goncharuk V. N. Diwan A. H. Zhang P. S. Shen S. S. Prieto V. G. (2005). Loss of claudin-1 expression in tumor-associated vessels correlates with acquisition of metastatic phenotype in melanocytic neoplasms. *J. Cutan. Pathol.* 32 533–536. 10.1111/j.0303-6987.2005.00324.x 16115050

[B25] Conesa A. Madrigal P. Tarazona S. Cervera A. McPherson A. Szczesniak M. W. (2016). A survey of best practices for RNA-seq data analysis. *Genome Biol.* 17:13. 10.1186/s13059-016-0881-8 26813401 PMC4728800

[B26] Cook D. J. Nguyen C. Chun H. N. Llorente L. I. Chiu A. S. Machnicki M. (2017). Hydrogel-delivered brain-derived neurotrophic factor promotes tissue repair and recovery after stroke. *J. Cereb. Blood Flow Metab.* 37 1030–1045. 10.1177/0271678X16649964 27174996 PMC5363479

[B27] Corrales L. Ajona D. Rafail S. Lasarte J. J. Riezu-Boj J. I. Lambris J. D. (2012). Anaphylatoxin C5a creates a favorable microenvironment for lung cancer progression. *J. Immunol.* 189 4674–4683. 10.4049/jimmunol.1201654 23028051 PMC3478398

[B28] De P. Peng Q. Traktuev D. O. Li W. Yoder M. C. March K. L. (2009). Expression of RAC2 in endothelial cells is required for the postnatal neovascular response. *Exp. Cell Res.* 315 248–263. 10.1016/j.yexcr.2008.10.003 19123268 PMC2767303

[B29] DeVita V. T. Lawrence T. S. Rosenberg S. A. (2012). *Cancer: Principles & Practice of Oncology: Primer of the Molecular Biology of Cancer*. Philadelphia, PA: Lippincott Williams & Wilkins.

[B30] Ding L. Kim M. Kanchi K. L. Dees N. D. Lu C. Griffith M. (2014). Clonal architectures and driver mutations in metastatic melanomas. *PLoS One* 9:e111153. 10.1371/journal.pone.0111153 25393105 PMC4230926

[B31] Ekström E. J. Sherwood V. Andersson T. (2011). Methylation and loss of secreted frizzled-related protein 3 enhances melanoma cell migration and invasion. *PLoS One* 6:e18674. 10.1371/journal.pone.0018674 21494614 PMC3072980

[B32] Ergul A. Alhusban A. Fagan S. C. (2012). Angiogenesis: a harmonized target for recovery after stroke. *Stroke* 43 2270–2274. 10.1161/STROKEAHA.111.642710 22618382 PMC3404267

[B33] Fanjul-Fernández M. Quesada V. Cabanillas R. Cadiñanos J. Fontanil T. Obaya A. (2013). Cell-cell adhesion genes CTNNA2 and CTNNA3 are tumour suppressors frequently mutated in laryngeal carcinomas. *Nat. Commun.* 4:2531. 10.1038/ncomms3531 24100690

[B34] Farooqi A. A. Tang J. Y. Li R. N. Ismail M. Chang Y. T. Shu C. W. (2015). Epigenetic mechanisms in cancer: push and pull between kneaded erasers and fate writers. *Int. J. Nanomed.* 10 3183–3191. 10.2147/IJN.S82527 25995628 PMC4425311

[B35] Forget M. A. Turcotte S. Beauseigle D. Godin-Ethier J. Pelletier S. Martin J. (2007). The Wnt pathway regulator DKK1 is preferentially expressed in hormone-resistant breast tumours and in some common cancer types. *Br. J. Cancer* 96 646–653. 10.1038/sj.bjc.6603579 17245340 PMC2360041

[B36] Fukumura D. Duda D. G. Munn L. L. Jain R. K. (2010). Tumor microvasculature and microenvironment: novel insights through intravital imaging in pre-clinical models. *Microcirculation* 17 206–225. 10.1111/j.1549-8719.2010.00029.x 20374484 PMC2859831

[B37] Gialeli C. Theocharis A. D. Karamanos N. K. (2011). Roles of matrix metalloproteinases in cancer progression and their pharmacological targeting. *FEBS J.* 278 16–27. 10.1111/j.1742-4658.2010.07919.x 21087457

[B38] Halaban R. Krauthammer M. Pelizzola M. Cheng E. Kovacs D. Sznol M. (2009). Integrative analysis of epigenetic modulation in melanoma cell response to decitabine: clinical implications. *PLoS One* 4:e4563. 10.1371/journal.pone.0004563 19234609 PMC2642998

[B39] Hansen T. M. Moss A. J. Brindle N. P. (2008). Vascular endothelial growth factor and angiopoietins in neurovascular regeneration and protection following stroke. *Curr. Neurovasc. Res.* 5 236–245. 10.2174/156720208786413433 18991658

[B40] Harik N. Harik S. I. Kuo N. T. Sakai K. Przybylski R. J. LaManna J. C. (1996). Time-course and reversibility of the hypoxia-induced alterations in cerebral vascularity and cerebral capillary glucose transporter density. *Brain Res.* 737 335–338. 10.1016/0006-8993(96)00965-1 8930387

[B41] Hassel J. C. Winnemöller D. Schartl M. Wellbrock C. (2008). STAT5 contributes to antiapoptosis in melanoma. *Melanoma Res.* 18 378–385. 10.1097/CMR.0b013e32830ce7d7 19011510

[B42] Herner A. Sauliunaite D. Michalski C. W. Erkan M. De Oliveira T Abiatari I. (2011). Glutamate increases pancreatic cancer cell invasion and migration via AMPA receptor activation and Kras-MAPK signaling. *Int. J. Cancer* 129 2349–2359. 10.1002/ijc.25898 21207374

[B43] Hillion J. A. Takahashi K. Maric D. Ruetzler C. Barker J. L. Hallenbeck J. M. (2005). Development of an ischemic tolerance model in a PC12 cell line. *J. Cereb. Blood Flow Metab.* 25 154–162. 10.1038/sj.jcbfm.9600003 15647748 PMC1378216

[B44] Ho H. Aruri J. Kapadia R. Mehr H. White M. A. Ganesan A. K. (2012). RhoJ regulates melanoma chemoresistance by suppressing pathways that sense DNA damage. *Cancer Res.* 72 5516–5528. 10.1158/0008-5472.CAN-12-0775 22971344 PMC3548429

[B45] Izraely S. Klein A. Meshel T. Tsarfaty G. Nahmias C. Couraud P. O. (2012). The metastatic microenvironment: brain-residing melanoma metastasis and dormant micrometastasis. *Int. J. Cancer* 131 1071–1082. 10.1002/ijc.27324 22025079

[B46] Izraely S. Klein A. Sagi-Assif O. Meshel T. Tsarfaty G. Hoon D. S. (2010). Chemokine-chemokine receptor axes in melanoma brain metastasis. *Immunol. Lett.* 130 107–114. 10.1016/j.imlet.2009.12.003 20005902

[B47] Izraely S. Sagi-Assif O. Klein A. Meshel T. Ben-Menachem S. Zaritsky A. (2015). The metastatic microenvironment: claudin-1 suppresses the malignant phenotype of melanoma brain metastasis. *Int. J. Cancer* 136 1296–1307. 10.1002/ijc.29090 25046141

[B48] Jiang X. Zhou J. Giobbie-Hurder A. Wargo J. Hodi F. S. (2013). The activation of MAPK in melanoma cells resistant to BRAF inhibition promotes PD-L1 expression that is reversible by MEK and PI3K inhibition. *Clin. Cancer Res.* 19 598–609. 10.1158/1078-0432.CCR-12-2731 23095323

[B49] Joshi S. Singh A. R. Zulcic M. Bao L. Messer K. Ideker T. (2014). Rac2 controls tumor growth, metastasis and M1-M2 macrophage differentiation in vivo. *PLoS One* 9:e95893. 10.1371/journal.pone.0095893 24770346 PMC4000195

[B50] Kesari S. Xiu J. Hu J. Michael E. S. Piccioni D. Reddy SK. (2015). Available at: https://www.carislifesciences.com/wp-content/uploads/2015/06/Tumor-profiles-of-brain-metastases-from-NSCLC-breast-cancer-and-melanoma_ASCO-2015.pdf (accessed 28 2017).

[B51] Kim J. Mori T. Chen S. L. Amersi F. F. Martinez S. R. Kuo C. (2006). Chemokine receptor CXCR4 expression in patients with melanoma and colorectal cancer liver metastases and the association with disease outcome. *Ann. Surg.* 244 113–120. 10.1097/01.sla.0000217690.65909.9c 16794396 PMC1570598

[B52] Klein A. Schwartz H. Meshel T. Izraely S. Bengaiev R. Nahmias C. (2015). Astrocytes facilitate melanoma brain metastasis via secretion of IL-23. *J. Pathol.* 236 116–127. 10.1002/path.4509 25639230

[B53] Klein-Goldberg A. Maman S. Witz I. P. (2013). The role played by the microenvironment in site-specific metastasis. *Cancer Lett.* 352 54–58. 10.1016/j.canlet.2013.08.029 23988268

[B54] Kostareli E. Holzinger D. Bogatyrova O. Hielscher T. Wichmann G. Keck M. (2013). HPV-related methylation signature predicts survival in oropharyngeal squamous cell carcinomas. *J. Clin. Invest.* 123 2488–2501. 10.1172/JCI67010 23635773 PMC3668826

[B55] Kuphal S. Lodermeyer S. Bataille F. Schuierer M. Hoang B. H. Bosserhoff A. K. (2006). Expression of *Dickkopf* genes is strongly reduced in malignant melanoma. *Oncogene* 25 5027–5036. 10.1038/sj.onc.1209508 16568085

[B56] Kwan D. H. Yung L. Y. Ye R. D. Wong Y. H. (2012). Activation of Ras-dependent signaling pathways by G(14) -coupled receptors requires the adaptor protein TPR1. *J. Cell Biochem.* 113 3486–3497. 10.1002/jcb.24225 22711498

[B57] Lamanna J. C. (2012). Angioplasticity and cerebrovascular remodeling. *Adv. Exp. Med. Biol.* 737 13–17. 10.1007/978-1-4614-1566-4_2 22259075

[B58] LaManna J. C. Kuo N. T. Lust W. D. (1998). Hypoxia-induced brain angiogenesis. Signals and consequences. *Adv. Exp. Med. Biol.* 454 287–293. 10.1007/978-1-4615-4863-8_34 9889903

[B59] Lange C. Storkebaum E. de Almodovar C. R. Dewerchin M. Carmeliet P. (2016). Vascular endothelial growth factor: a neurovascular target in neurological diseases. *Nat. Rev. Neurol.* 12 439–454. 10.1038/nrneurol.2016.88 27364743

[B60] Langley R. R. Fidler I. J. (2013). The biology of brain metastasis. *Clin. Chem.* 59 180–189. 10.1373/clinchem.2012.193342 23115057

[B61] Lee M. M. Chui R. K. Tam I. Y. Lau A. H. Wong Y. H. (2012). CCR1-mediated STAT3 tyrosine phosphorylation and CXCL8 expression in THP-1 macrophage-like cells involve pertussis toxin-insensitive Gα(14/16) signaling and IL-6 release. *J. Immunol.* 189 5266–5276. 10.4049/jimmunol.1103359 23125416

[B62] Leotlela P. D. Wade M. S. Duray P. H. Rhode M. J. Brown H. F. Rosenthal D. T. (2007). Claudin-1 overexpression in melanoma is regulated by PKC and contributes to melanoma cell motility. *Oncogene* 26 3846–3856. 10.1038/sj.onc.1210155 17160014

[B63] Li L. Dragulev B. Zigrino P. Mauch C. Fox J. W. (2009). The invasive potential of human melanoma cell lines correlates with their ability to alter fibroblast gene expression in vitro and the stromal microenvironment in vivo. *Int. J. Cancer* 125 1796–1804. 10.1002/ijc.24463 19569239

[B64] Li S. Nie E. H. Yin Y. Benowitz L. I. Tung S. Vinters H. V. (2015). GDF10 is a signal for axonal sprouting and functional recovery after stroke. *Nat. Neurosci.* 18 1737–1745. 10.1038/nn.4146 26502261 PMC4790086

[B65] Liao Z. Lutz J. Nevalainen M. T. (2010). Transcription factor Stat5a/b as a therapeutic target protein for prostate cancer. *Int. J. Biochem. Cell Biol.* 42 186–192. 10.1016/j.biocel.2009.11.001 19914392 PMC2818495

[B66] Liberzon A. Birger C. Thorvaldsdottir H. Ghandi M. Mesirov J. P. Tamayo P. (2015). The molecular signatures database (MSigDB) hallmark gene set collection. *Cell Syst.* 1 417–425. 10.1016/j.cels.2015.12.004 26771021 PMC4707969

[B67] Lin J. C. Ho W. H. Gurney A. Rosenthal A. (2003). The netrin-G1 ligand NGL-1 promotes the outgrowth of thalamocortical axons. *Nat. Neurosci.* 6 1270–1276. 10.1038/nn1148 14595443

[B68] Lindvall O. Kokaia Z. (2015). Neurogenesis following stroke affecting the adult brain. *Cold Spring Harb. Perspect. Biol.* 7:a019034. 10.1101/cshperspect.a019034 26525150 PMC4632663

[B69] Liu A. M. Wong Y. H. (2005). Activation of nuclear factor κB by somatostatin type 2 receptor in pancreatic acinar AR42J cells involves Gα14and multiple signaling components. *J. Biol. Chem.* 280 34617–34625. 10.1074/jbc.m504264200 16115892

[B70] Long F. Liu H. Hahn C. Sumazin P. Zhang M. Q. Zilberstein A. (2004). Genome-wide prediction and analysis of function-specific transcription factor binding sites. *In Silico Biol.* 4 395–410. 15506990

[B71] Lu Y. Xiong Y. Huo Y. Han J. Yang X. Zhang R. (2011). Grb-2-associated binder 1 (Gab1) regulates postnatal ischemic and VEGF-induced angiogenesis through the protein kinase A-endothelial NOS pathway. *Proc. Natl. Acad. Sci. U.S.A.* 108 2957–2962. 10.1073/pnas.1009395108 21282639 PMC3041066

[B72] Luksch H. Uckermann O. Stepulak A. Hendruschk S. Marzahn J. Bastian S. (2011). Silencing of selected glutamate receptor subunits modulates cancer growth. *Anticancer Res.* 31 3181–3192. 21965725

[B73] Lutsenko S. V. Kiselev S. M. Severin S. E. (2003). Molecular mechanisms of tumor angiogenesis. *Biochemistry* 68 286–300. 12733970 10.1023/a:1023002216413

[B74] Martin S. T. Sato N. Dhara S. Chang R. Hustinx S. R. Abe T. (2005). Aberrant methylation of the Human Hedgehog interacting protein (HHIP) gene in pancreatic neoplasms. *Cancer Biol. Ther.* 4 728–733. 10.4161/cbt.4.7.1802 15970691

[B75] Marzese D. M. Witz I. P. Kelly D. F. Hoon D. S. (2015). Epigenomic landscape of melanoma progression to brain metastasis: unexplored therapeutic alternatives. *Epigenomics* 7 1303–1311. 10.2217/epi.15.77 26638944

[B76] Mauldin I. S. Craig L. S. Wang E. (2014). TLR2/6 agonists and IFN-gamma treatment induces favorable immune cell recruiting signatures from melanoma associated with STAT1 and IL-32 signaling. *J. ImmunoTher. Cancer* 2(Suppl. 3):P225 10.1186/2051-1426-2-s3-p225

[B77] Mazon M. Masi D. Carreau M. (2016). Modulating dickkopf-1: a Strategy to Monitor or treat cancer? *Cancers* 8:E62. 10.3390/cancers8070062 27367730 PMC4963804

[B78] McDermott E. P. O’Neill L. A. (2002). Ras participates in the activation of p38 MAPK by interleukin-1 by associating with IRAK, IRAK2, TRAF6, and TAK-1. *J. Biol. Chem.* 277 7808–7815. 10.1074/jbc.M108133200 11744690

[B79] Micevic G. Theodosakis N. Bosenberg M. (2017). Aberrant DNA methylation in melanoma: biomarker and therapeutic opportunities. *Clin. Epigenet.* 9:34. 10.1186/s13148-017-0332-8 28396701 PMC5381063

[B80] Miettinen M. McCue P. A. Sarlomo-Rikala M. Rys J. Czapiewski P. Wazny K. (2014). GATA3: a multispecific but potentially useful marker in surgical pathology: a systematic analysis of 2500 epithelial and nonepithelial tumors. *Am. J. Surg. Pathol.* 38 13–22. 10.1097/PAS.0b013e3182a0218f 24145643 PMC3991431

[B81] Mirmohammadsadegh A. Hassan M. Bardenheuer W. Marini A. Gustrau A. Nambiar S. (2006). STAT5 phosphorylation in malignant melanoma is important for survival and is mediated through SRC and JAK1 kinases. *J. Invest. Dermatol.* 126 2272–2280. 10.1038/sj.jid.5700385 16741510

[B82] Morris D. C. Yeich T. Khalighi M. M. Soltanian-Zadeh H. Zhang Z. G. Chopp M. (2003). Microvascular structure after embolic focal cerebral ischemia in the rat. *Brain Res.* 972 31–37. 10.1016/S0006-8993(03)02433-8 12711075

[B83] Nabizadeh J. Akhir M. D. Rolfe B. E. (2013). “Role of the Complement system in melanoma tumour growth,” in *Proceedings of the 6th International Conference on Complement Therapeutics*, (Kos).

[B84] Nicholl M. B. Chen X. Qin C. Bai Q. Zhu Z. Davis M. R. (2016). IL-32α has differential effects on proliferation and apoptosis of human melanoma cell lines. *J. Surg. Oncol.* 113 364–369. 10.1002/jso.24142 27100023

[B85] Niessner H. Kosnopfel C. Sinnberg T. Beck D. Krieg K. Wanke I. (2017). Combined activity of temozolomide and the mTOR inhibitor temsirolimus in metastatic melanoma involves DKK1. *Exp. Dermatol.* 26 598–606. 10.1111/exd.13372 28423208

[B86] Nitta H. Wada Y. Kawano Y. Murakami Y. Irie A. Taniguchi K. (2013). Enhancement of human cancer cell motility and invasiveness by anaphylatoxin C5a via aberrantly expressed C5a receptor (CD88). *Clin. Cancer Res.* 19 2004–2013. 10.1158/1078-0432.CCR-12-1204 23287562

[B87] Noel A. Jost M. Maquoi E. (2008). Matrix metalloproteinases at cancer tumor-host interface. *Semin. Cell Dev. Biol.* 19 52–60. 10.1016/j.semcdb.2007.05.011 17625931

[B88] Ohab J. J. Carmichael S. T. (2008). Poststroke neurogenesis: emerging principles of migration and localization of immature neurons. *Neuroscientist* 14 369–380. 10.1177/1073858407309545 18024854

[B89] Ohab J. J. Fleming S. Blesch A. Carmichael S. T. (2006). A neurovascular niche for neurogenesis after stroke. *J. Neurosci.* 26 13007–13016. 10.1523/JNEUROSCI.4323-06.2006 17167090 PMC6674957

[B90] Olsen C. L. Hsu P. P. Glienke J. Rubanyi G. M. Brooks A. R. (2004). Hedgehog-interacting protein is highly expressed in endothelial cells but down-regulated during angiogenesis and in several human tumors. *BMC Cancer* 4:43. 10.1186/1471-2407-4-43 15294024 PMC512291

[B91] Oren M. (2003). Decision making by p53: life, death and cancer. *Cell Death Differ.* 10 431–442. 10.1038/sj.cdd.4401183 12719720

[B92] Oshima H. Ishikawa T. Yoshida G. J. Naoi K. Maeda Y. Naka K. (2014). TNF-α/TNFR1 signaling promotes gastric tumorigenesis through induction of Noxo1 and Gna14 in tumor cells. *Oncogene* 33 3820–3829. 10.1038/onc.2013.356 23975421

[B93] Ozgur A. Vu T. Erkan G. Radev D. R. (2008). Identifying gene-disease associations using centrality on a literature mined gene-interaction network. *Bioinformatics* 24 i277–i285. 10.1093/bioinformatics/btn182 18586725 PMC2718658

[B94] Palmieri D. ed (2012). “An introduction to brain metastasis,”. *Central Nervous System Metastasis, the Biological Basis and Clinical Considerations* (Springer: Heidelberg) 10.1007/978-94-007-5291-7

[B95] Prakash R. Carmichael S. T. (2015). Blood-brain barrier breakdown and neovascularization processes after stroke and traumatic brain injury. *Curr. Opin. Neurol.* 28 556–564. 10.1097/WCO.0000000000000248 26402408 PMC5267616

[B96] Psaila B. Lyden D. (2009). The metastatic niche: adapting the foreign soil. *Nat. Rev. Cancer* 9 285–293. 10.1038/nrc2621 19308068 PMC3682494

[B97] Qian C. N. Tan M. H. Yang J. P. Cao Y. (2016). Revisiting tumor angiogenesis: vessel co-option, vessel remodeling, and cancer cell-derived vasculature formation. *Chin. J. Cancer* 35 10. 10.1186/s40880-015-0070-2 26747273 PMC4706692

[B98] Quintana E. Piskounova E. Shackleton M. Weinberg D. Eskiocak U. Fullen D. R. (2012). Human melanoma metastasis in NSG mice correlates with clinical outcome in patients. *Sci. Transl. Med.* 4:159ra149. 10.1126/scitranslmed.3004599 23136044 PMC4501487

[B99] Raso A. Mascelli S. Biassoni R. Nozza P. Kool M. Pistorio A. (2011). High levels of PROM1 (CD133) transcript are a potential predictor of poor prognosis in medulloblastoma. *Neuro. Oncol.* 13 500–508. 10.1093/neuonc/nor022 21486962 PMC3093338

[B100] Risso D. Ngai J. Speed T. P. Dudoit S. (2014). Normalization of RNA-seq data using factor analysis of control genes or samples. *Nat. Biotechnol.* 32 896–902. 10.1038/nbt.2931 25150836 PMC4404308

[B101] Rivals I. Personnaz L. Taing L. Potier M. C. (2007). Enrichment or depletion of a GO category within a class of genes: which test? *Bioinformatics* 23 401–407. 10.1093/bioinformatics/btl633 17182697

[B102] Robinson M. D. McCarthy D. J. Smyth G. K. (2010). edgeR: a bioconductor package for differential expression analysis of digital gene expression data. *Bioinformatics* 26 139–140. 10.1093/bioinformatics/btp616 19910308 PMC2796818

[B103] Rochman Y. Kashyap M. Robinson G. W. Sakamoto K. Gomez-Rodriguez J. Wagner K. U. (2010). Thymic stromal lymphopoietin-mediated STAT5 phosphorylation via kinases JAK1 and JAK2 reveals a key difference from IL-7-induced signaling. *Proc. Natl. Acad. Sci. U.S.A.* 107 19455–19460. 10.1073/pnas.1008271107 20974963 PMC2984176

[B104] Rollins D. (2016). *Netrin G1 in Desmoplastic Fibroblasts Enhances Interactions with Pancreatic Cancer Cells (Order No. 10154210)*. Available at: https://search.proquest.com/docview/1836084588?accountid=14512 (accessed 28, 2017).

[B105] Salmaggi A. Maderna E. Calatozzolo C. Gaviani P. Canazza A. Milanesi I. (2009). CXCL12, CXCR4 and CXCR7 expression in brain metastases. *Cancer Biol. Ther.* 8 1608–1614. 10.4161/cbt.8.17.9202 19625779

[B106] Sang H. Li T. Li H. Liu J. (2015). Gab1 regulates proliferation and migration through the PI3K/Akt signaling pathway in intrahepatic cholangiocarcinoma. *Tumour Biol.* 36 8367–8377. 10.1007/s13277-015-3590-0 26014518

[B107] Sarris E. G. Saif M. W. Syrigos K. N. (2012). The biological role of PI3K pathway in lung cancer. *Pharmaceuticals* 5 1236–1264. 10.3390/ph5111236 24281308 PMC3816662

[B108] Savaskan N. E. Seufert S. Hauke J. Tränkle C. Eyüpoglu I. Y. Hahnen E. (2011). Dissection of mitogenic and neurodegenerative actions of cystine and glutamate in malignant gliomas. *Oncogene* 30 43–53. 10.1038/onc.2010.391 20802520

[B109] Schmidt J. W. Wehde B. L. Sakamoto K. Triplett A. A. Anderson S. M. Tsichlis P. N. (2014). Stat5 regulates the phosphatidylinositol 3-kinase/Akt1 pathway during mammary gland development and tumorigenesis. *Mol. Cell. Biol.* 34 1363–1377. 10.1128/MCB.01220-13 24469394 PMC3993568

[B110] Seiden-Long I. Navab R. Shih W. Li M. Chow J. Zhu C. Q. (2008). Gab1 but not Grb2 mediates tumor progression in Met overexpressing colorectal cancer cells. *Carcinogenesis* 29 647–655. 10.1093/carcin/bgn009 18192688

[B111] Sharfman W. H. (2012). “Immunotherapies for metastatic melanoma,” in *Melanoma (emerging cancer therapeutics)*, eds Sharfman W. H. Arbraham J. (New York, NY: Demos Medical Publishing).

[B112] Shen L. Gao Y. Qian J. Wu Y. Zhou M. Sun A. (2012). The role of SDF-1α/Rac pathway in the regulation of endothelial progenitor cell polarity; homing and expression of Rac1, Rac2 during endothelial repair. *Mol. Cell. Biochem.* 365 1–7. 10.1007/s11010-011-1083-z 21964561

[B113] Shepelev M. V. Korobko I. V. (2012). Pak6 protein kinase is a novel effector of an atypical Rho family GTPase Chp/RhoV. *Biochemistry* 77 26–32. 10.1134/S0006297912010038 22339630

[B114] Shi L. Wang K. Zhao M. Yuan X. Huang C. (2010a). Overexpression of PIP5KL1 suppresses the growth of human cervical cancer cells in vitro and in vivo. *Cell Biol. Int.* 34 309–315. 10.1042/CBI20090040 19947914

[B115] Shi L. Zhao M. Luo Q. Ma Y. M. Zhong J. L. Yuan X. H. (2010b). Overexpression of PIP5KL1 suppresses cell proliferation and migration in human gastric cancer cells. *Mol. Biol. Rep.* 37 2189–2198. 10.1007/s11033-009-9701-5 19680787

[B116] Sho S. Court C. M. Winograd P. Russell M. M. Tomlinson J. S. (2017). A prognostic mutation panel for predicting cancer recurrence in stages II and III colorectal cancer. *J. Surg. Oncol.* 116 996–1004. 10.1002/jso.24781 28767131

[B117] Soneson C. Love M. I. Robinson M. D. (2015). Differential analyses for RNA-seq: transcript-level estimates improve gene-level inferences. *F1000Res.* 4:1521. 10.12688/f1000research.7563.2 26925227 PMC4712774

[B118] Song Y. Ori-McKenney K. M. Zheng Y. Han C. Jan L. Y. Jan Y. N. (2012). Regeneration of drosophila sensory neuron axons and dendrites is regulated by the Akt pathway involving Pten and microRNA bantam. *Genes Dev.* 26 1612–1625. 10.1101/gad.193243.112 22759636 PMC3404388

[B119] Springer M. L. Ip T. K. Blau H. M. (2000). Angiogenesis monitored by perfusion with a space-filling microbead suspension. *Mol. Ther.* 1 82–87. 10.1006/mthe.1999.0006 10933915

[B120] Stecca B. Mas C. Clement V. Zbinden M. Correa R. Piguet V. (2007). Melanomas require HEDGEHOG-GLI signaling regulated by interactions between GLI1 and the RAS-MEK/AKT pathways. *Proc. Natl. Acad. Sci. U.S.A.* 104 5895–5900. 10.1073/pnas.0700776104 17392427 PMC1838820

[B121] Subramanian A. Tamayo P. Mootha V. K. Mukherjee S. Ebert B. L. Gillette M. A. (2005). Gene set enrichment analysis: a knowledge-based approach for interpreting genome-wide expression profiles. *Proc. Natl. Acad. Sci. U.S.A.* 102 15545–15550. 10.1073/pnas.0506580102 16199517 PMC1239896

[B122] Sun J. He H. Pillai S. Xiong Y. Challa S. Xu L. (2013). GATA3 transcription factor abrogates Smad4 transcription factor-mediated fascin overexpression, invadopodium formation, and breast cancer cell invasion. *J. Biol. Chem.* 288 36971–36982. 10.1074/jbc.M113.506535 24235142 PMC3873555

[B123] Takaku M. Grimm S. A. Wade P. A. (2015). GATA3 in breast cancer: tumor suppressor or oncogene? *Gene Expr.* 16 163–168. 10.3727/105221615x14399878166113 26637396 PMC4758516

[B124] Tang Y. Li M. Wang J. Pan Y. Wu F. X. (2015). CytoNCA: a cytoscape plugin for centrality analysis and evaluation of protein interaction networks. *Biosystems* 127 67–72. 10.1016/j.biosystems.2014.11.005 25451770

[B125] Tarnawski A. S. Ahluwalia A. Jones M. K. (2014). Angiogenesis in gastric mucosa: an important component of gastric erosion and ulcer healing and its impairment in aging. *J Gastroenterol. Hepatol.* 29(Suppl. 4) 112–123. 10.1111/jgh.12734 25521743

[B126] Tenreiro M. M. Ferreira R. Bernardino L. Brito M. A. (2016). Cellular response of the blood-brain barrier to injury: potential biomarkers and therapeutic targets for brain regeneration. *Neurobiol. Dis.* 91 262–273. 10.1016/j.nbd.2016.03.014 26996728

[B127] Termini J. Neman J. Jandial R. (2014). Role of the neural niche in brain metastatic cancer. *Cancer Res.* 74 4011–4015. 10.1158/0008-5472.CAN-14-1226 25035392 PMC4122250

[B128] Tomer R. Ye L. Hsueh B. Deisseroth K. (2014). Advanced CLARITY for rapid and high-resolution imaging of intact tissues. *Nat. Protoc.* 9 1682–1697. 10.1038/nprot.2014.123 24945384 PMC4096681

[B129] van Zijl F. Krupitza G. Mikulits W. (2011). Initial steps of metastasis: cell invasion and endothelial transmigration. *Mutat. Res.* 728 23–34. 10.1016/j.mrrev.2011.05.002 21605699 PMC4028085

[B130] Vasyutina E. Stebler J. Brand-Saberi B. Schulz S. Raz E. Birchmeier C. (2005). CXCR4 and Gab1 cooperate to control the development of migrating muscle progenitor cells. *Genes Dev.* 19 2187–2198. 10.1101/gad.346205 16166380 PMC1221889

[B131] Venkatesh H. S. Johung T. B. Caretti V. Noll A. Tang Y. Nagaraja S. (2015). Neuronal activity promotes glioma growth through neuroligin-3 secretion. *Cell* 161 803–816. 10.1016/j.cell.2015.04.012 25913192 PMC4447122

[B132] Wang L. Gao X. Gao P. Deng W. Yu P. Ma J. (2006). Cell-based screening and validation of human novel genes associated with cell viability. *J. Biomol. Screen.* 11 369–376. 10.1177/1087057106286654 16751333

[B133] Wang S. Fan W. Wan B. Tu M. Jin F. Liu F. (2017). Characterization of long noncoding RNA and messenger RNA signatures in melanoma tumorigenesis and metastasis. *PLoS One* 12:e0172498. 10.1371/journal.pone.0172498 28225791 PMC5321451

[B134] Weidle U. H. Birzele F. Kollmorgen G. Krüger A. (2016). Molecular targets and pathways involved in liver metastasis of colorectal cancer. *Clin. Exp. Metastasis* 32 623–635. 10.1007/s10585-015-9732-3 26104118

[B135] Weitzenfeld P. Ben-Baruch A. (2013). The chemokine system, and its CCR5 and CXCR4 receptors, as potential targets for personalized therapy in cancer. *Cancer Lett.* 352 36–53. 10.1016/j.canlet.2013.10.006 24141062

[B136] Wen S. J. Zhang W. Ni N. N. Wu Q. Wang X. P. Lin Y. K. (2017). Expression of Rho GTPases family in melanoma cells and its influence on cytoskeleton and migration. *Oncotarget* 8 30112–30122. 10.18632/oncotarget.15618 28404912 PMC5444730

[B137] Wikman H. Lamszus K. Detels N. Uslar L. Wrage M. Benner C. (2012). Relevance of PTEN loss in brain metastasis formation in breast cancer patients. *Breast Cancer Res.* 14:R49. 10.1186/bcr3150 22429330 PMC3446383

[B138] Yan G. N. Yang L. Lv Y. F. Shi Y. Shen L. L. Yao X. H. (2014). Endothelial cells promote stem-like phenotype of glioma cells through activating the hedgehog pathway. *J. Pathol.* 234 11–22. 10.1002/path.4349 24604164 PMC4260128

[B139] Yang B. Treweek J. B. Kulkarni R. P. Deverman B. E. Chen C. K. Lubeck E. (2014). Single-cell phenotyping within transparent intact tissue through whole-body clearing. *Cell* 158 945–958. 10.1016/j.cell.2014.07.017 25088144 PMC4153367

[B140] Yang J. P. Liu H. J. Liu X. F. (2010). VEGF promotes angiogenesis and functional recovery in stroke rats. *J. Invest. Surg.* 23 149–155. 10.3109/08941930903469482 20590386

[B141] Yang L. Huang J. Ren X. Gorska A. E. Chytil A. Aakre M. (2008). Abrogation of TGF beta signaling in mammary carcinomas recruits Gr-1+CD11b+ myeloid cells that promote metastasis. *Cancer Cell* 13 23–35. 10.1016/j.ccr.2007.12.004 18167337 PMC2245859

[B142] Yang L. Shah K. K. Abbruscato T. J. (2012). An in vitro model of ischemic stroke. *Methods Mol. Biol.* 814 451–466. 10.1007/978-1-61779-452-0_30 22144325

[B143] Yao Y. Ye H. Qi Z. Mo L. Yue Q. Baral A. (2016). B7-H4(B7x)-mediated cross-talk between glioma-initiating cells and macrophages via the IL6/JAK/STAT3 pathway lead to poor prognosis in glioma patients. *Clin. Cancer Res.* 22 2778–2790. 10.1158/1078-0432.CCR-15-0858 27001312 PMC4891287

[B144] Yao C. Su L. Shan J. Zhu C. Liu L. Liu C. (2016). IGF/STAT3/NANOG/Slug signaling axis simultaneously controls epithelial-mesenchymal transition and stemness maintenance in colorectal cancer. *Stem Cells* 34 820–831. 10.1002/stem.2320 26840943

[B145] Zhang L. Zhang S. Yao J. Lowery F. J. Zhang Q. Huang W. C. (2015). Microenvironment-induced PTEN loss by exosomal microRNA primes brain metastasis outgrowth. *Nature* 527 100–104. 10.1038/nature15376 26479035 PMC4819404

[B146] Zhang Y. (2012). STAT5a-targeting miRNA enhances chemosensitivity to cisplatin and 5-fluorouracil in human colorectal cancer cells. *Mol. Med. Rep.* 5 1215–1219. 10.3892/mmr.2012.801 22367509

[B147] Zhang Z. Liu F. Li Z. Wang D. Li R. Sun C. (2017). Jak3 is involved in CCR7-dependent migration and invasion in metastatic squamous cell carcinoma of the head and neck. *Oncol. Lett.* 13 3191–3197. 10.3892/ol.2017.5861 28521425 PMC5431255

[B148] Zhao J. Wang W. Ha C. H. Kim J. Y. Wong C. Redmond E. M. (2011). Endothelial Grb2-associated binder 1 is crucial for postnatal angiogenesis. *Arterioscler. Thromb. Vasc. Biol.* 31 1016–1023. 10.1161/ATVBAHA.111.224493 21372298 PMC3094153

[B149] Zi X. Guo Y. Simoneau A. R. Hope C. Xie J. Holcombe R. F. (2005). Expression of Frzb/secreted frizzled-related protein 3, a secreted Wnt antagonist, in human androgen-independent prostate cancer PC-3 cells suppresses tumor growth and cellular invasiveness. *Cancer Res.* 65 9762–9770. 10.1158/0008-5472.CAN-05-0103 16266997

[B150] Ziyad S. Iruela-Arispe M. L. (2011). Molecular mechanisms of tumor angiogenesis. *Genes Cancer* 2 1085–1096. 10.1177/1947601911432334 22866200 PMC3411131

