# Supplementary figures and images for: Regeneration Enhances Metastasis: A Novel Role for Neurovascular Signaling in Promoting Melanoma Brain Metastasis
Source: Front Neurosci. 2019 Apr 9;13:297. doi: 10.3389/fnins.2019.00297 (PMC6465799; doi:10.3389/fnins.2019.00297)

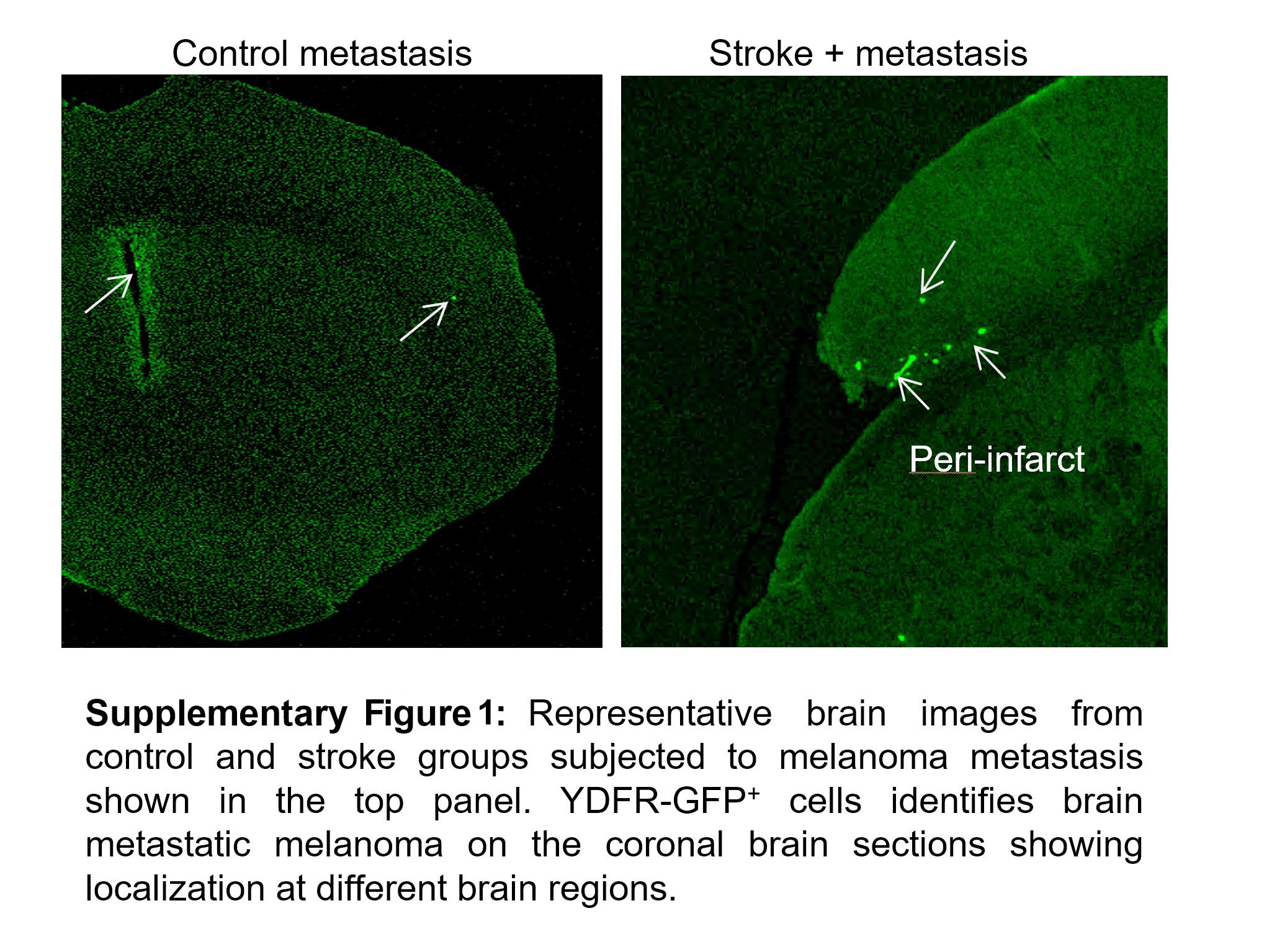

Supplement: Supplementary file 2 [file Image_1.jpg]
